# Spousal concordance and interdependence in depression among older adults: A cross-cultural longitudinal study in China, the United States, and England

**DOI:** 10.1017/S0033291726105042

**Published:** 2026-07-29

**Authors:** Jinfeng Miao, Ruhan Zhu, Mingzhu Huang, Shujuan Gan, Zhiting Chen, Yanmin Wu, Jianhua Guan, Jintao Chen, Bingbo Zhuang, Hanrong Dong, Yuxin Xu, Min Zhang, Yuanxiao Li, Jianmin Liu, Wenhuo Chen, Tingyu Yi

**Affiliations:** 1 Fujian Medical University Union Hospital, China; 2 Zhangzhou Affiliated Hospital of Fujian Medical University, China

**Keywords:** aging cohorts, cross-cultural comparison, depression interdependence, functional limitations, random-intercept cross-lagged panel model, spousal concordance

## Abstract

**Background:**

Late-life depression often extends to the marital partner, yet mechanisms of dyadic interdependence and their cross-cultural generalizability remain unclear.

**Methods:**

We analyzed harmonized longitudinal data from couples in the China Health and Retirement Longitudinal Study (CHARLS; *n* = 3,532; 5 waves, 2011–2020), the Health and Retirement Study (HRS; *n* = 2,332; 8 waves, 2006–2020), and the English Longitudinal Study of Ageing (ELSA; *n* = 1,895; 9 waves, 2002–2018). Depressive symptoms (CES-D) and activities of daily living (ADL)/instrumental ADL (IADL) limitations were measured at every wave. Complementary actor–partner interdependence models (APIM) and random-intercept cross-lagged panel models (RI-CLPM) were applied, with multiplicity adjustment, full-information maximum likelihood (FIML), and 20-imputation triangulation.

**Results:**

Within-couple correlations of depressive symptoms were positive in every cohort (*r* = 0.34–0.43 in CHARLS, 0.14–0.20 in HRS, 0.23–0.29 in ELSA). APIM partner effects were robust in CHARLS but sparse elsewhere. After partialling out stable between-couple similarity, RI-CLPM within-person cross-spouse associations were attenuated, reaching consistent significance under FIML and multiple imputation only in ELSA. Four-variable RI-CLPMs showed bidirectional depression–disability coupling, strongest in HRS and ELSA, with cross-spouse function-to-depression effects in Western cohorts but not in CHARLS. Sex distinguishability was not supported, and half-longitudinal mediation through ADL/IADL did not survive multiplicity correction.

**Conclusions:**

Concordance in late-life depressive symptoms replicates across three major aging cohorts; in CHARLS, it primarily reflects stable between-couple similarity, whereas modest within-person prospective coupling persists in ELSA and in the multiple-imputation HRS estimates. Bidirectional depression–disability coupling in Western cohorts is the most replicable within-person signal, supporting combined depression management and functional rehabilitation for older couples.

## Introduction

Depression in later life is a major source of suffering and excess disability, with pooled population-based surveys placing the prevalence of clinically significant depressive symptoms at approximately 35.1% (95% CI 30.2%–40.4%) among community-dwelling adults aged 60 years and older (Cai et al., 2023). The Global Burden of Disease Study 2021 documented a substantial and rising global burden of late-life depression, with marked inequities across countries and sociodemographic strata and a heavier burden in women than in men (Sun et al., 2025), and the broader 2019 study reported a global rise in mental disorder disability‑adjusted life years (DALYs) from 80.8 million in 1990 to 125.3 million in 2019 (GBD, 2022 Mental Disorders Collaborators, 2022). Late-life depression is closely associated with functional decline, social withdrawal, and elevated mortality. Although it has historically been studied as an individual-level disorder, depressive symptoms in older adults are deeply embedded in the marital relationship. Married and cohabiting partners show systematic similarities in depressive symptoms, a phenomenon termed spousal concordance (Meyler, Stimpson, & Peek, 2007), and recent large-scale evidence indicates that having a depressed partner substantially elevates an older adult’s own risk of depression (Han et al., 2023). These observations have motivated growing interest in late-life depression as a fundamentally dyadic phenomenon (Kiecolt-Glaser & Wilson, 2017).

Three mechanisms have been proposed to account for spousal concordance in depressive symptoms: assortative mating on shared dispositional and socioeconomic characteristics (Horwitz, Balbona, Paulich, & Keller, 2023); cumulative joint exposure to common environments and life stressors; and dynamic interpersonal influence on mood through empathy, daily interaction, and emotional contagion. A factor that operates within all three is physical functioning. At the individual level, depressive symptoms predict subsequent decline in activities of daily living (ADL) and instrumental ADL (IADL) (Penninx et al., 1998), and the reverse direction has been documented in large Chinese longitudinal cohorts (Liu et al., 2023; Zhou, Wang, & Ma, 2024; Zhu et al., 2024). Whether this individual-level bidirectional pattern persists in a dyadic specification that partials out stable between-couple similarity, however, remains to be tested. Within couples, one partner’s functional decline can also increase caregiving demands on the other, a mechanism formalized by the stress-process model as a contributor to elevated depressive symptoms in the caregiving spouse (Pearlin, Mullan, Semple, & Skaff, 1990).

The magnitude of spousal interdependence may vary across cultural contexts. China, the United States, and England offer informative contrasts in family structure and caregiving norms: spouses are the principal caregivers of disabled older adults in the United States and Europe, whereas adult children continue to provide most long-term care in China (Pinquart & Sörensen, 2011). One of the few harmonized cross-national comparisons of older couples to date reported stronger spousal concordance in depressive symptoms in China than in the United States or England (Lu & Shelley, 2019); a recent three-cohort analysis of China Health and Retirement Longitudinal Study (CHARLS), Health and Retirement Study (HRS), and SHARE similarly found that adverse-childhood-experience–depression spousal concordance is stronger in China than in Western settings (Sun et al., 2023). Most existing dyadic studies of late-life depression have relied on the actor–partner interdependence model (APIM), which does not separate stable between-couple similarity from prospective within-person change (Cook & Kenny, 2005; Hamaker, Kuiper, & Grasman, 2015; Kenny, Kashy, & Cook, 2006). A two-wave APIM analysis of 1,245 spousal caregiving dyads in CHARLS (Liu et al., 2023) reported gender-asymmetric partner effects, but its generalizability beyond a Chinese caregiving subsample remains untested. Recent extensions of the random-intercept cross-lagged panel model to dyadic data (Mund, Park, & Nestler, 2024) provide a means to disentangle between-couple stability from prospective within-person change in mixed-sex couples, but applications to late-life depression across socioculturally distinct cohorts remain scarce. Whether the prospective coupling between depressive symptoms and functional limitations operates consistently within and across spouses in different sociocultural settings, and whether observed concordance reflects dynamic interpersonal influence or stable shared characteristics, therefore remains unresolved.

Building on these gaps, we examined the dyadic interdependence of depressive symptoms and functional limitations in older couples using harmonized longitudinal data from CHARLS, HRS, and English Longitudinal Study of Ageing (ELSA) (Sonnega et al., 2014; Steptoe, Breeze, Banks, & Nazroo, 2013; Zhao et al., 2014). We applied complementary APIM and random-intercept cross-lagged panel models to characterize within-couple depressive concordance, prospective within-person cross-spouse associations, and bidirectional within- and cross-spouse coupling between depressive symptoms and ADL/IADL limitations across three socioculturally and demographically distinct cohorts.

## Methods

### Study design and data sources

This longitudinal dyadic study examined the prospective interdependence of depressive symptoms and functional limitations in older couples using harmonized data from three population-representative aging cohorts: CHARLS, HRS, and ELSA (Sonnega et al., 2014; Steptoe et al., 2013; Zhao et al., 2014). The three cohorts share comparable two-stage probability sampling, biennial or triennial follow-up, and harmonized core measures of mental and physical health, enabling cross-national dyadic analysis. We analyzed five CHARLS waves (2011, 2013, 2015, 2018, 2020), eight HRS waves (2006–2020), and nine ELSA waves (2002–2018).

### Participants

Eligible participants were married or partnered couples in which both spouses contributed at least three valid waves of depression assessments over the observation window. Couples were identified through household identifiers, retaining households containing exactly two adults reporting being married or partnered with each other. Same-sex couples were retained in the descriptive within-couple similarity analyses and in the sex-stratified individual-level analyses, with each individual assigned to the male or female stratum according to their own sex; because the dyadic APIM and random-intercept cross-lagged panel model (RI-CLPM) specifications require exactly one male partner and one female partner per couple, however, those structural models are by construction restricted to mixed-sex couples. At the individual level, we excluded participants who were younger than 50 years or had missing age, who had a documented history of psychiatric illness, or who had a documented history of dementia. Individuals whose coresident spouse was removed under any of these criteria, and who therefore no longer had a partner available for dyadic analysis, were also excluded. After applying these criteria, the analytic samples comprised 3,532 couples in CHARLS, 2,332 couples in HRS, and 1,895 couples in ELSA. Of the 1,895 ELSA couples, 1,891 contributed to the structural equation models, the four-couple difference reflecting lavaan’s full-information maximum likelihood (FIML) coverage threshold for model-implied covariance estimation; CHARLS and HRS samples were unaffected at this stage.

### Measures

Depressive symptoms were assessed with two well-validated short forms of the Center for Epidemiologic Studies Depression Scale: the 10-item form (CES-D-10) in CHARLS (Andresen, Malmgren, Carter, & Patrick, 1994; Boey, 1999) and the 8-item form (CES-D-8) in HRS and ELSA (Kohout, Berkman, Evans, & Cornoni-Huntley, 1993). Each cohort’s score was standardized using the baseline-pooled mean and standard deviation across both spouses, and depressive symptoms were additionally dichotomized at the conventional clinical thresholds (CES-D-10 ≥ 10; CES-D-8 ≥ 3) so that core models could be re-estimated with linear-probability and logistic specifications as a clinically interpretable bridge across the two short forms.

Functional limitations were measured with two harmonized inventories of identical length across the three cohorts: a six-item ADL scale and a five-item IADL scale. Within each cohort and at each wave, the ADL and IADL indicators were constructed as the unweighted count of items endorsed as ‘has any difficulty’, yielding integer scores ranging from 0 to 6 for ADL and 0 to 5 for IADL. No items were dropped, and no differential weighting was applied. Both indicators were z-standardized within the cohort to the baseline-pooled distribution. Spousal care was operationalized as a four-level categorical variable: care received from spouse, care needed but not received, no care needed, and other. Covariates were age and educational attainment at baseline (time-invariant) and self-rated health, which was treated as a time-varying covariate measured at the predictor wave of each transition. All three covariates were measured at the individual level for both spouses; in dyadic models, both the actor’s and the partner’s values at the corresponding wave were included.

### Statistical analysis

We applied a complementary analytic framework combining the APIM, the bivariate RI-CLPM, and a four-variable RI-CLPM that jointly modeled both spouses’ depression and functional limitations (Cook & Kenny, 2005; Hamaker et al., 2015; Mulder & Hamaker, 2021; Mund et al., 2024). The APIM yields wave-by-wave actor and partner effects on the within-cohort z-score scale and supports dyadic-pattern classification through the Kenny–Ledermann *k* parameter and the partner-to-actor effect ratio with a delta-method 95% confidence interval (Kenny & Ledermann, 2010). The RI-CLPM decomposes each repeated score into a person-specific random intercept and a wave-specific within-person residual, restricting all cross-lagged paths to the within-person residuals and thereby separating stable between-couple similarity from dynamic within-person change (Hamaker et al., 2015). Reporting followed the STROBE guidelines for observational studies; the completed checklist is provided as Supplementary File S1.

Wave-by-wave APIMs predicted each spouse’s depressive-symptom z-score at wave *t* + 1 from both spouses’ z-scores at wave *t*, adjusting for both spouses’ age, education, baseline ADL and IADL counts, and self-rated health (with self-rated health measured at the predictor wave). Sex distinguishability was tested at each transition by a joint Wald test on role-by-actor and role-by-partner interactions in long-format dyadic regressions with cluster-robust standard errors. Care-moderated APIMs and prespecified subgroup analyses (by mean couple age and husband’s education) used formal partner-by-moderator interaction tests.

The RI-CLPM was specified in two nested forms: a bivariate two-variable model containing both spouses’ depression at all waves, and a multivariate four-variable model jointly including both spouses’ depression and functional status. The substantive focus comprised eight pre-specified within-person cross-construct cross-lags linking each spouse’s own and partner’s depression and functional status across consecutive waves (Penninx et al., 1998; Zhu et al., 2024). The four-variable model was fitted with ADL as the primary functional indicator and re-fitted with IADL as a sensitivity check; nested model comparisons used the Satorra–Bentler scaled-difference chi-squared test (Satorra & Bentler, 2010). To address state-dependent reporting bias in self-reported function, each model was re-estimated under a specification fixing all four depression-to-function paths at zero, and the resulting function-to-depression paths were compared with the bidirectional estimates. All RI-CLPMs were estimated in lavaan with robust maximum likelihood and FIML for missingness (Rosseel, 2012). Sex symmetry at the structural level was assessed using joint Wald tests on the eight key cross-lags.

Longitudinal mediation followed Cole and Maxwell’s (2003) three-wave half-longitudinal framework, with predictor at wave *t*, mediator at wave *t* + 1, and outcome at wave *t* + 2, adjusting for the outcome spouse’s baseline depression and both spouses’ baseline functional status, age, education, and actor self-rated health. Four primary *a priori* pathways (each spouse’s depression to own ADL or IADL to the other spouse’s depression) were tested with bias-corrected and percentile bootstrap 95% confidence intervals (1,000 replicates; Preacher & Hayes, 2008); partner-mediator and cross-construct pathways were treated as exploratory.

In parallel with FIML, we generated *m* = 20 multiple imputations in Python with a Bayesian-ridge IterativeImputer and pooled estimates by Rubin’s rules, reporting the fraction of missing information for each pooled coefficient (van Buuren & Groothuis-Oudshoorn, 2011). The missing-data mechanism was examined before model fitting: Little’s test rejected the missing-completely-at-random null in all three cohorts, and logistic regressions of final-wave dropout on both spouses’ baseline depressive symptoms, functional limitations, self-rated health, age, and education showed that attrition was systematically related to observed baseline characteristics, consistent with a missing-at-random mechanism (Supplementary Table S9). Multiplicity was controlled within hypothesis families: Holm correction for primary hypotheses (Holm, 1979) and Benjamini–Hochberg false discovery rate (FDR) control for secondary hypotheses (Benjamini & Hochberg, 1995). The two-sided alpha level was 0.05 throughout.

As a psychometric supplement, we assessed cross-cohort measurement invariance of the CES-D by multigroup confirmatory factor analysis (MGCFA) on the six items administered in common across CHARLS, HRS, and ELSA, fitted in lavaan with the WLSMV estimator for binary indicators (Rosseel, 2012). Configural, full metric, and partial metric models were compared using the Cheung–Rensvold criteria (Cheung & Rensvold, 2002), with the CHARLS Likert responses dichotomized at two thresholds (≥3 and ≥2) to verify robustness.

## Results

### Sample characteristics

Baseline characteristics are summarized in Table 1. The analytic sample comprised 3,532 CHARLS couples followed across five waves (2011–2020), 2,332 HRS couples across eight waves (2006–2020), and 1,895 ELSA couples across nine waves (2002–2018). Husbands were on average 2–3 years older than their wives in every cohort (paired *t*-test, all *p* < 0.001), and the cohorts differed markedly in age structure, education, and functional burden. CHARLS couples were the youngest and least educated (85.8% of husbands and 93.6% of wives below high-school level) but had the highest baseline ADL/IADL limitations; HRS couples were the oldest (38.9% of husbands aged ≥70 years) yet had the lowest functional burden; ELSA couples were intermediate in age but had the highest prevalence of spousal-care receipt (12.2% of husbands and 20.2% of wives). At every wave in every cohort, wives reported higher CES-D sum scores than their husbands (paired *t*-test, *p* < 0.05 across all 22 wave-by-cohort cells).

**Table 1.** Sample characteristics and baseline distributional indicators in the three cohorts Table 1. Long description.

Characteristic	CHARLS (*n* = 3,532; CES-D-10, 0–30)			HRS (*n* = 2,332; CES-D-8, 0–8)			ELSA (*n* = 1,895; CES-D-8, 0–8)		
	Husbands	Wives	*p*	Husbands	Wives	*p*	Husbands	Wives	*p*
Age, mean ± SD (years)	61.3 ± 6.7	59.3 ± 6.1	<0.001	67.0 ± 8.4	64.0 ± 8.2	<0.001	63.6 ± 8.3	61.3 ± 7.9	<0.001
50–59, *n* (%)	1,636 (46.3)	2,057 (58.2)	—	517 (22.2)	794 (34.0)	—	703 (37.1)	905 (47.8)	—
60–69, *n* (%)	1,439 (40.7)	1,223 (34.6)	—	908 (38.9)	924 (39.6)	—	713 (37.6)	664 (35.0)	—
70–79, *n* (%)	429 (12.1)	243 (6.9)	—	727 (31.2)	532 (22.8)	—	410 (21.6)	293 (15.5)	—
≥80, *n* (%)	28 (0.8)	9 (0.3)	—	180 (7.7)	82 (3.5)	—	69 (3.6)	33 (1.7)	—
Education level, *n* (%)			<0.001			<0.001			<0.001
Low (below high school)	3,031 (85.8)	3,305 (93.6)	—	433 (18.6)	325 (13.9)	—	534 (29.6)	721 (43.0)	—
Medium (high school)	427 (12.1)	207 (5.9)	—	1,159 (49.8)	1,435 (61.6)	—	916 (50.7)	756 (45.1)	—
High (college +)	74 (2.1)	20 (0.6)	—	735 (31.6)	570 (24.5)	—	357 (19.8)	198 (11.8)	—
***CES-D sum score, mean ± SD (per wave)***									
W1 (2011/2006/2002)	7.1 ± 5.7	9.2 ± 6.4	<0.001	0.8 ± 1.3	1.0 ± 1.5	<0.001	0.9 ± 1.4	1.3 ± 1.7	<0.001
W2 (2013/2008/2004)	6.8 ± 5.1	8.6 ± 5.9	<0.001	0.8 ± 1.3	1.0 ± 1.5	<0.001	1.0 ± 1.4	1.4 ± 1.7	<0.001
W3 (2015/2010/2006)	6.8 ± 5.7	8.9 ± 6.5	<0.001	0.8 ± 1.3	1.0 ± 1.5	<0.001	0.9 ± 1.4	1.3 ± 1.7	<0.001
W4 (2018/2012/2008)	7.4 ± 5.9	9.3 ± 6.6	<0.001	0.8 ± 1.4	0.9 ± 1.5	0.035	0.8 ± 1.4	1.2 ± 1.7	<0.001
W5 (2020/2014/2010)	7.7 ± 5.9	10.0 ± 6.5	<0.001	0.8 ± 1.4	1.0 ± 1.5	0.002	1.0 ± 1.5	1.3 ± 1.7	<0.001
W6 (HRS 2016/ELSA 2012)	—	—	—	0.8 ± 1.4	0.9 ± 1.5	0.019	0.7 ± 1.3	1.2 ± 1.7	<0.001
W7 (HRS 2018/ELSA 2014)	—	—	—	0.8 ± 1.4	0.9 ± 1.5	0.037	0.8 ± 1.3	1.2 ± 1.6	<0.001
W8 (HRS 2020/ELSA 2016)	—	—	—	0.8 ± 1.4	1.1 ± 1.7	<0.001	0.8 ± 1.2	1.3 ± 1.6	<0.001
W9 (ELSA 2018)	—	—	—	—	—	—	0.8 ± 1.2	1.3 ± 1.6	<0.001
Baseline ADL (0–6), mean ± SD	0.24 ± 0.78	0.35 ± 0.92	<0.001	0.12 ± 0.49	0.16 ± 0.61	0.028	0.28 ± 0.77	0.24 ± 0.78	0.107
Baseline IADL (0–5), mean ± SD	0.29 ± 0.82	0.46 ± 1.01	<0.001	0.07 ± 0.34	0.07 ± 0.36	0.865	0.07 ± 0.35	0.11 ± 0.43	0.012
Baseline self-rated health, *n* (%)			<0.001			0.038			0.392
Excellent	258 (7.3)	166 (4.7)	—	64 (2.7)	67 (2.9)	—	306 (16.3)	308 (16.3)	—
Very good	633 (18.0)	500 (14.2)	—	336 (14.4)	303 (13.0)	—	619 (32.9)	616 (32.7)	—
Good	1,825 (51.8)	1,714 (48.6)	—	750 (32.2)	679 (29.1)	—	581 (30.9)	623 (33.1)	—
Fair	691 (19.6)	968 (27.5)	—	837 (35.9)	893 (38.3)	—	292 (15.5)	269 (14.3)	—
Poor	118 (3.3)	178 (5.0)	—	342 (14.7)	389 (16.7)	—	84 (4.5)	68 (3.6)	—
***Baseline spousal-care status, n (%)***									
Care received from spouse	302 (8.6)	396 (11.2)	—	50 (2.1)	61 (2.6)	—	232 (12.2)	382 (20.2)	—
Care needed but not received	460 (13.0)	669 (18.9)	—	216 (9.3)	189 (8.1)	—	172 (9.1)	123 (6.5)	—
No care needed	2,762 (78.2)	2,460 (69.7)	—	2,066 (88.6)	2,082 (89.3)	—	1,479 (78.0)	1,379 (72.8)	—
Other	8 (0.2)	7 (0.2)	—	0 (0.0)	0 (0.0)	—	12 (0.6)	11 (0.6)	—

*Note:* Values are mean ± SD for continuous variables and *n* (%) for categorical variables. *p*-values compare husbands vs. wives within each cohort: paired *t*-test for continuous variables and χ^2^ test for categorical variables (em-dash indicates that the test is reported only at the variable level, not at each category). Em dashes in CES-D wave rows denote that the corresponding wave does not exist in that cohort’s sampling design (CHARLS has 5 waves, HRS has 8 waves, ELSA has 9 waves). CES-D, Center for Epidemiologic Studies Depression Scale; CHARLS uses the 10-item version (4-point Likert; sum-score range 0–30; clinical cut-off ≥10), HRS and ELSA use the 8-item version (binary; sum-score range 0–8; clinical cut-off ≥3). ADL, activities of daily living (range 0–6); IADL, instrumental activities of daily living (range 0–5). Counts and percentages are based on observed (non-imputed) baseline data; for categorical variables, percentages are computed among participants with non-missing data on that variable, so category counts may not sum to the cohort total where a baseline item was incompletely recorded. Missing data were handled by full-information maximum likelihood and multiple imputation in all inferential analyses.

### Within-couple similarity and actor–partner interdependence models

Standardized CES-D scores were positively correlated within couples at every wave in every cohort, with magnitudes that differed substantially across populations: Pearson *r* = 0.343–0.432 in CHARLS, 0.139–0.204 in HRS, and 0.234–0.288 in ELSA (all *p* < 0.001; Supplementary Table S1).

Cross-cohort MGCFA on the six common CES-D items supported configural invariance (comparative fit index [CFI] = 0.981, root mean square error of approximation [RMSEA] = 0.052, standardised root mean square residual [SRMR] = 0.046) and established partial metric invariance with the positively keyed ‘Happy’ item loading freely estimated (CFI = 0.979, ΔCFI = −0.003 vs. configural, RMSEA = 0.048), meeting the Cheung–Rensvold criteria under both binarization thresholds. Five of the six items showed closely matched loadings across cohorts (cross-cohort range 0.065–0.135), whereas the ‘Happy’ item loading varied substantially (CHARLS = 0.380; ELSA = 0.707; HRS = 0.783). The latent depression construct is therefore comparable across cohorts for structural-coefficient comparison, while quantitative comparison of latent means remains bounded; full numerical detail is reported in Supplementary Table S10.

Wave-by-wave APIMs (Table 2) showed large and statistically significant actor effects across all three cohorts (*β* = 0.326–0.575; all *p* FDR < 0.001), reflecting strong intra-personal continuity of depressive symptoms over 2-year intervals. Partner effects were more heterogeneous: in CHARLS, seven of eight transitions yielded a partner effect that survived Benjamini–Hochberg correction (*β* = 0.053–0.107 for the surviving cells), whereas only 2 of 14 HRS transitions and 1 of 16 ELSA transitions did so (the latter being the W4 → W5 wife outcome, *β* = 0.115, *p* FDR = 0.012). The Kenny–Ledermann *k* parameter was uniformly below unity (median 0.169, 0.066, and 0.099 in CHARLS, HRS, and ELSA, respectively), and most cells were classified as actor-only or mixed. These wave-wise patterns were reproduced both by multiple-imputation–pooled estimates (*m* = 20; Supplementary Table S2) and by a binary clinical-cutoff sensitivity analysis (CES-D-10 ≥ 10 in CHARLS; CES-D-8 ≥ 3 in HRS and ELSA), with cross-scale partner-effect agreement of 87.5%, 78.6%, and 100% in CHARLS, HRS, and ELSA, respectively (linear probability model; logistic-model agreement of 87.5%, 78.6%, and 93.8%; Supplementary Table S2 Panel B).

**Table 2.** Wave-by-wave actor–partner interdependence model (APIM) estimates, Kenny–Ledermann *k* parameter, and dyadic-pattern classification in the three cohorts (listwise-deletion analysis) Table 2. Long description.

Transition	Outcome	*n*	Actor β (SE)	*p* FDR	Partner β (SE)	*p* FDR	*k*	95% CI for k	Pattern
***CHARLS***									
W1 → W2 (2011–13)	M	2,750	0.354 (0.018)	<0.001	0.061 (0.015)	< 0.001	0.173	(0.080, 0.267)	Mixed
W1 → W2 (2011–13)	F	2,750	0.371 (0.019)	<0.001	0.072 (0.020)	< 0.001	0.195	(0.082, 0.309)	Mixed
W2 → W3 (2013–15)	M	2,788	0.432 (0.021)	<0.001	0.070 (0.018)	< 0.001	0.162	(0.076, 0.248)	Mixed
W2 → W3 (2013–15)	F	2,788	0.444 (0.021)	<0.001	0.075 (0.023)	0.001	0.169	(0.063, 0.274)	Mixed
W3 → W4 (2015–18)	M	2,556	0.435 (0.020)	<0.001	0.074 (0.018)	< 0.001	0.169	(0.084, 0.254)	Mixed
W3 → W4 (2015–18)	F	2,556	0.419 (0.021)	<0.001	0.107 (0.022)	< 0.001	0.254	(0.142, 0.367)	Mixed
W4 → W5 (2018–20)	M	2,008	0.398 (0.022)	<0.001	0.036 (0.019)	0.061	0.090	(−0.007, 0.188)	Actor-only
W4 → W5 (2018–20)	F	2,008	0.443 (0.022)	<0.001	0.053 (0.024)	0.030	0.120	(0.010, 0.229)	Mixed
***HRS***									
W1 → W2 (2006–08)	M	2,121	0.485 (0.021)	<0.001	−0.005 (0.017)	0.759	−0.011	(−0.079, 0.058)	Actor-only
W1 → W2 (2006–08)	F	2,121	0.397 (0.021)	<0.001	0.047 (0.024)	0.168	0.119	(−0.001, 0.239)	Mixed
W2 → W3 (2008–10)	M	2,097	0.459 (0.019)	<0.001	0.039 (0.016)	0.077	0.085	(0.015, 0.155)	Mixed
W2 → W3 (2008–10)	F	2,097	0.367 (0.021)	<0.001	0.070 (0.022)	0.025	0.191	(0.067, 0.315)	Mixed
W3 → W4 (2010–12)	M	1,869	0.469 (0.025)	<0.001	0.009 (0.020)	0.759	0.019	(−0.065, 0.103)	Actor-only
W3 → W4 (2010–12)	F	1,869	0.376 (0.021)	<0.001	0.008 (0.024)	0.759	0.020	(−0.107, 0.147)	Actor-only
W4 → W5 (2012–14)	M	1,615	0.513 (0.022)	<0.001	0.031 (0.021)	0.370	0.061	(−0.019, 0.141)	Actor-only
W4 → W5 (2012–14)	F	1,615	0.411 (0.025)	<0.001	0.030 (0.025)	0.381	0.074	(−0.044, 0.193)	Actor-only
W5 → W6 (2014–16)	M	1,312	0.499 (0.027)	<0.001	0.008 (0.022)	0.759	0.016	(−0.072, 0.105)	Actor-only
W5 → W6 (2014–16)	F	1,312	0.431 (0.026)	<0.001	0.038 (0.029)	0.377	0.088	(−0.045, 0.222)	Actor-only
W6 → W7 (2016–18)	M	1,028	0.537 (0.029)	<0.001	−0.020 (0.026)	0.614	−0.038	(−0.134, 0.058)	Actor-only
W6 → W7 (2016–18)	F	1,028	0.507 (0.032)	<0.001	0.091 (0.032)	0.032	0.180	(0.053, 0.306)	Mixed
W7 → W8 (2018–20)	M	808	0.553 (0.035)	<0.001	0.039 (0.030)	0.377	0.071	(−0.035, 0.177)	Actor-only
W7 → W8 (2018–20)	F	808	0.575 (0.038)	<0.001	−0.033 (0.041)	0.614	−0.057	(−0.198, 0.084)	Actor-only
***ELSA***									
W1 → W2 (2002–04)	M	1,471	0.374 (0.026)	<0.001	0.006 (0.020)	0.861	0.017	(−0.090, 0.123)	Actor-only
W1 → W2 (2002–04)	F	1,471	0.375 (0.025)	<0.001	0.017 (0.031)	0.812	0.044	(−0.116, 0.204)	Actor-only
W2 → W3 (2004–06)	M	1,421	0.388 (0.025)	<0.001	−0.012 (0.020)	0.812	−0.031	(−0.130, 0.068)	Actor-only
W2 → W3 (2004–06)	F	1,421	0.408 (0.025)	<0.001	0.048 (0.030)	0.239	0.119	(−0.026, 0.264)	Actor-only
W3 → W4 (2006–08)	M	1,171	0.488 (0.028)	<0.001	0.053 (0.022)	0.051	0.108	(0.019, 0.196)	Mixed
W3 → W4 (2006–08)	F	1,171	0.416 (0.027)	<0.001	0.091 (0.034)	0.051	0.218	(0.054, 0.382)	Mixed
W4 → W5 (2008–10)	M	1,041	0.460 (0.031)	<0.001	0.011 (0.025)	0.812	0.024	(−0.083, 0.130)	Actor-only
W4 → W5 (2008–10)	F	1,041	0.386 (0.030)	<0.001	0.115 (0.034)	0.012	0.297	(0.119, 0.476)	Mixed
W5 → W6 (2010–12)	M	959	0.326 (0.028)	<0.001	0.060 (0.023)	0.051	0.183	(0.040, 0.326)	Mixed
W5 → W6 (2010–12)	F	959	0.443 (0.029)	<0.001	0.016 (0.033)	0.812	0.035	(−0.110, 0.181)	Actor-only
W6 → W7 (2012–14)	M	832	0.432 (0.035)	<0.001	0.004 (0.026)	0.884	0.009	(−0.108, 0.126)	Actor-only
W6 → W7 (2012–14)	F	832	0.471 (0.033)	<0.001	0.091 (0.042)	0.079	0.192	(0.017, 0.368)	Mixed
W7 → W8 (2014–16)	M	690	0.438 (0.035)	<0.001	0.039 (0.028)	0.289	0.089	(−0.036, 0.214)	Actor-only
W7 → W8 (2014–16)	F	690	0.451 (0.037)	<0.001	0.011 (0.044)	0.861	0.024	(−0.167, 0.215)	Actor-only
W8 → W9 (2016–18)	M	566	0.496 (0.045)	<0.001	0.082 (0.033)	0.051	0.164	(0.030, 0.299)	Mixed
W8 → W9 (2016–18)	F	566	0.448 (0.045)	<0.001	0.091 (0.059)	0.239	0.204	(−0.055, 0.463)	Actor-only

*Note:* Each row reports a wave-to-wave APIM in which the depressive-symptom z-score of the outcome spouse at wave *t* + 1 is regressed on both spouses’ z-scores at wave *t*, adjusting for actor age, education, and baseline functional status (own and partner ADL, IADL, and own self-rated health). Actor β and partner β are unstandardized regression coefficients on the standardized depression metric. *p* FDR is the Benjamini–Hochberg-adjusted *p*-value across the cohort-internal tests per outcome. The Kenny–Ledermann *k* parameter is the partner-to-actor effect ratio with a delta-method 95% CI; the pattern label reflects the three-test classification (couple-pattern, actor-only, mixed) using unadjusted *p* < 0.05 thresholds. F, female outcome; M, male outcome.

### Longitudinal mediation through ADL/IADL and care-receipt moderation

Three-wave half-longitudinal mediation models (Supplementary Table S3) yielded no indirect (*ab*) pathway through ADL or IADL that survived Holm’s correction within the *a priori* primary family in any cohort, with bias-corrected indirect effects uniformly small (|*ab*| < 0.013). Three wife-mediated pathways from wife to husband depression approached uncorrected significance: two through wife IADL in CHARLS at W1–W3 (*ab* = 0.0040, *p* = 0.014) and W3–W5 (*ab* = 0.0052, *p* = 0.008), and one through wife ADL in ELSA at W5–W7 (*ab* = 0.0124, *p* = 0.012); none retained significance after multiplicity adjustment. In care-moderated APIMs (Supplementary Table S4), the joint Wald *F* test of the partner × care-category interactions was nonsignificant at every transition in CHARLS and ELSA after Benjamini–Hochberg correction; only two HRS female-outcome transitions reached *p* < 0.05 (W5 → W6 *F* = 5.93; W6 → W7 *F* = 7.32).

### Bivariate random-intercept cross-lagged panel model

The time-invariant Hamaker bivariate RI-CLPM fitted the data well in all three cohorts (CFI 0.963–0.988; RMSEA 0.033–0.036; SRMR 0.032–0.057; Supplementary Table S5), whereas the traditional cross-lagged panel model (CLPM) that conflates between- and within-person variance fitted poorly (CFI 0.761–0.813; RMSEA 0.088–0.124). At the between-person level, the standardized correlations between husband and wife random intercepts were uniformly large and highly significant (CHARLS *ρ* = 0.523; ELSA *ρ* = 0.416; HRS *ρ* = 0.305; all *p* < 0.001).

**Figure 1.**
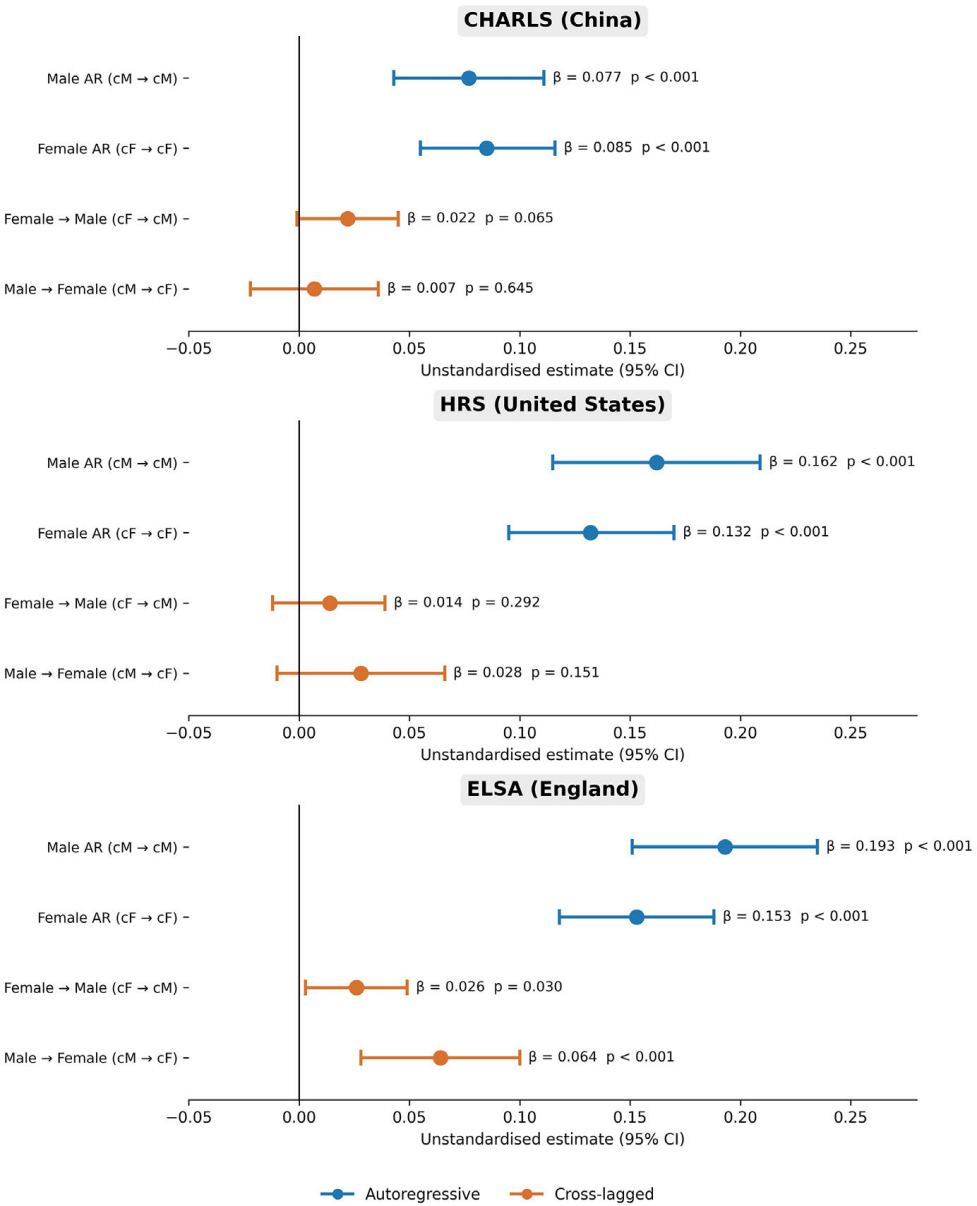
Time-invariant within-person cross-lagged paths from the bivariate random-intercept cross-lagged panel model, by cohort. *Note:* Forest plot of the four time-invariant within-person paths estimated by the Hamaker et al. (2015) bivariate random-intercept cross-lagged panel model, separately by cohort. cM = within-person component of the male partner’s standardized CES-D score; cF = the analogous female component. The two autoregressive paths (Male AR: cM → cM; Female AR: cF → cF) and two cross-spouse cross-lagged paths (Male → Female: cM → cF; Female → Male: cF → cM) are reported as unstandardized estimates with 95% confidence intervals. Estimates were obtained by full-information maximum likelihood with robust (MLR) standard errors; the figure displays the FIML estimates, and the multiple-imputation-pooled estimates pooled by Rubin’s rules are reported in Supplementary Table S6. The cross-spouse paths are statistically distinguishable from zero only in ELSA (Male → Female *β* = 0.064, *p* < 0.001; Female → Male *β* = 0.026, *p* = 0.030); they are not distinguishable from zero in CHARLS or HRS under FIML. Full model parameters appear in Supplementary Table S5. Figure 1. Long description.

Once this stable similarity was partialled out, within-person cross-lagged transmission (Figure 1) was small in all three cohorts and reached significance only in ELSA. Under FIML, husband-to-wife and wife-to-husband cross-lags were not distinguishable from zero in CHARLS (*x*MF *β* = 0.007, *p* = 0.645; *x*FM *β* = 0.022, *p* = 0.065) or HRS (*x*MF *β* = 0.028, *p* = 0.151; *x*FM *β* = 0.014, *p* = 0.292) but were both significant in ELSA (*x*MF *β* = 0.064, *p* < 0.001; *x*FM *β* = 0.026, *p* = 0.030). Multiple-imputation estimates pooled by Rubin’s rules (Supplementary Table S6) yielded significant cross-spouse paths in HRS (*x*MF *β* = 0.061; *x*FM *β* = 0.044; both *p* < 0.001) and reinforced the ELSA findings (*x*MF *β* = 0.087; *x*FM *β* = 0.051; both *p* < 0.001), but remained nonsignificant in CHARLS.

### Four-variable random-intercept cross-lagged panel model and reporting-bias robustness

**Figure 2.**
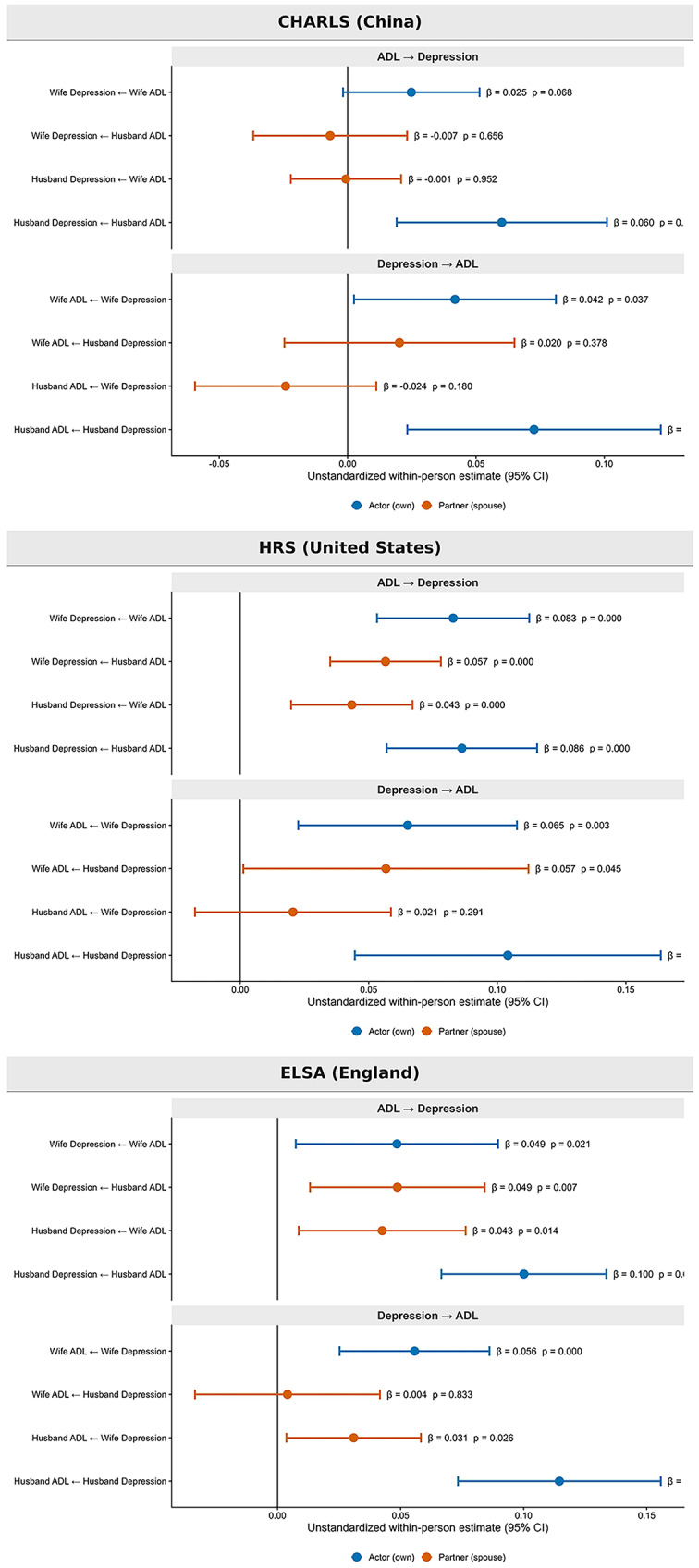
Eight key within-person cross-construct cross-lags from the four-variable random-intercept cross-lagged panel model (depressive symptoms × ADL count), primary specification, by cohort. *Note:* For each of the three cohorts, eight time-invariant within-person cross-construct paths from the four-variable RI-CLPM jointly modeling depressive symptoms and ADL functional limitations are plotted as standardized estimates with 95% confidence intervals. The paths are organized by direction (function → depression and depression → function) and by role (actor: own predictor → own outcome; partner: spouse predictor → own outcome) and stratified by sex (F, female outcome; M, male outcome). Solid-filled markers denote estimates significant at *p* < 0.05; open markers denote non-significant estimates. The full coefficient table (*β*, SE, 95% CI, *p*, standardized *β*) is given in Table 3. Reporting-bias robustness analyses (IADL sensitivity, Satorra–Bentler scaled difference test, and bivariate-vs.-four-variable attenuation diagnostic) are reported in Supplementary Table S8. Figure 2. Long description.

Joint Wald tests of sex distinguishability did not reject the symmetric-dyad null at any wave transition in any cohort after Benjamini–Hochberg correction (all *p* > 0.10; Supplementary Table S7), supporting the indistinguishable-dyad specification adopted for the multivariate model. The four-variable RI-CLPM jointly modeled depression and ADL counts for both spouses (Table 3; Figure 2). Within-person actor effects of own functional limitations on own subsequent depressive symptoms were positive and significant in HRS (husband *β* = 0.086; wife *β* = 0.083; both *p* < 0.001) and ELSA (husband *β* = 0.100, *p* < 0.001; wife *β* = 0.049, *p* = 0.021); in CHARLS, only the husband actor path was statistically significant (*β* = 0.060, *p* = 0.004; wife *β* = 0.025, *p* = 0.068). Partner effects of one spouse’s functional limitations on the other spouse’s subsequent depressive symptoms were significant in both Western cohorts (HRS: husband outcome *β* = 0.043, wife outcome *β* = 0.057, both *p* < 0.001; ELSA: husband *β* = 0.043, *p* = 0.014; wife *β* = 0.049, *p* = 0.007) but were not distinguishable from zero in CHARLS. IADL substitution produced effects of comparable direction and magnitude (Supplementary Table S8), and the within-person depression–depression cross-lags from the bivariate model were not materially modified by adding functional limitations (overlapping 95% confidence intervals in all eight depression-to-depression paths in each cohort).

**Table 3.** Four-variable random-intercept cross-lagged panel model (ADL primary mechanism model): time-invariant within-person cross-construct paths between depressive symptoms and ADL functional status, by cohort Table 3. Long description.

Path	Description	Outcome ← Predictor	*β* (SE)	95% CI	*p*	Std. *β*	Sig.
***CHARLS***							
bMdMf	Actor: own function → own depression	Male depression ← Male ADL	0.060 (0.021)	[0.019, 0.101]	0.004	0.077	*
bMdFf	Partner: spouse function → own depression	Male depression ← Female ADL	−0.001 (0.011)	[−0.022, 0.021]	0.952	−.001	
bMfMd	Actor: own depression → own function	Male ADL ← Male depression	0.073 (0.025)	[0.023, 0.122]	0.004	0.050	*
bMfFd	Partner: spouse depression → own function	Male ADL ← Female depression	−0.024 (0.018)	[−0.059, 0.011]	0.180	−.019	
bFdMf	Partner: spouse function → own depression	Female depression ← Male ADL	−0.007 (0.015)	[−0.037, 0.023]	0.656	−.008	
bFdFf	Actor: own function → own depression	Female depression ← Female ADL	0.025 (0.014)	[−0.002, 0.051]	0.068	0.031	
bFfMd	Partner: spouse depression → own function	Female ADL ← Male depression	0.020 (0.023)	[−0.025, 0.065]	0.378	0.013	
bFfFd	Actor: own depression → own function	Female ADL ← Female depression	0.042 (0.020)	[0.002, 0.081]	0.037	0.031	*
***HRS***							
bMdMf	Actor: own function → own depression	Male depression ← Male ADL	0.086 (0.015)	[0.057, 0.115]	<0.001	0.153	***
bMdFf	Partner: spouse function → own depression	Male depression ← Female ADL	0.043 (0.012)	[0.020, 0.067]	<0.001	0.081	***
bMfMd	Actor: own depression → own function	Male ADL ← Male depression	0.104 (0.030)	[0.045, 0.164]	<0.001	0.052	***
bMfFd	Partner: spouse depression → own function	Male ADL ← Female depression	0.021 (0.019)	[−0.018, 0.059]	0.291	0.012	
bFdMf	Partner: spouse function → own depression	Female depression ← Male ADL	0.057 (0.011)	[0.035, 0.078]	<0.001	0.087	***
bFdFf	Actor: own function → own depression	Female depression ← Female ADL	0.083 (0.015)	[0.053, 0.112]	<0.001	0.133	***
bFfMd	Partner: spouse depression → own function	Female ADL ← Male depression	0.057 (0.028)	[0.001, 0.112]	0.045	0.027	*
bFfFd	Actor: own depression → own function	Female ADL ← Female depression	0.065 (0.022)	[0.023, 0.108]	0.003	0.036	**
***ELSA***							
bMdMf	Actor: own function → own depression	Male depression ← Male ADL	0.100 (0.017)	[0.067, 0.134]	<0.001	0.130	***
bMdFf	Partner: spouse function → own depression	Male depression ← Female ADL	0.043 (0.017)	[0.009, 0.076]	0.014	0.051	*
bMfMd	Actor: own depression → own function	Male ADL ← Male depression	0.115 (0.021)	[0.073, 0.156]	<0.001	0.083	***
bMfFd	Partner: spouse depression → own function	Male ADL ← Female depression	0.031 (0.014)	[0.004, 0.058]	0.026	0.020	*
bFdMf	Partner: spouse function → own depression	Female depression ← Male ADL	0.049 (0.018)	[0.013, 0.084]	0.007	0.064	**
bFdFf	Actor: own function → own depression	Female depression ← Female ADL	0.049 (0.021)	[0.007, 0.090]	0.021	0.057	*
bFfMd	Partner: spouse depression → own function	Female ADL ← Male depression	0.004 (0.019)	[−0.034, 0.042]	0.833	0.003	
bFfFd	Actor: own depression → own function	Female ADL ← Female depression	0.056 (0.016)	[0.025, 0.086]	<0.001	0.041	***

*Note:* Time-invariant within-person paths from the four-variable RI-CLPM jointly modelling depressive symptoms and ADL functional limitations within the same Hamaker et al. (2015) random-intercept structure, with cross-spouse symmetry assumed (indistinguishable-dyad specification, justified by Supplementary Table S7). *β*, unstandardized regression coefficient (with cluster-robust SE) on the within-cohort z-standardized depression metric and the raw 0–6 ADL count; Std. *β*, completely standardized solution; Sig.: *p < 0.05; **p < 0.01; ***p < 0.001 (within-model, unadjusted). d, depression; f, function (ADL); F, female partner; M, male partner. The eight key cross-construct paths comprise four function-to-depression paths (bMdMf, bMdFf, bFdMf, bFdFf) and four depression-to-function paths (bMfMd, bMfFd, bFfMd, bFfFd). Per-path comparison of bidirectional and forward-only specifications and IADL sensitivity model results are reported in Supplementary Table S8.

As a robustness check against possible state-dependent reporting bias in self-rated function, we refitted each four-variable model with the four depression-to-function paths fixed at zero. Satorra–Bentler scaled difference tests rejected this constraint in five of six cohort × functional-indicator combinations (all *p* ≤ 0.002, full statistics in Supplementary Table S8); only CHARLS IADL was compatible with the constraint (*p* = 0.180). The bidirectional specification was therefore preferred. Critically, the function-to-depression coefficients were themselves robust to this specification choice: across all four function-to-depression paths in each of the three cohorts of the ADL primary model, 95% confidence intervals from the bidirectional and constrained models overlapped, and actor point estimates attenuated by only 19–42% under the constrained specification.

## Discussion

In a harmonized longitudinal analysis of 7,759 older couples followed for up to 16 years across three nationally representative aging cohorts, three principal findings emerged. First, within-couple concordance of depressive symptoms was replicated in every cohort but was substantially stronger in CHARLS (Pearson *r* = 0.34–0.43) than in HRS (*r* = 0.14–0.20) or ELSA (*r* = 0.23–0.29). Second, after partialling out stable between-couple similarity in the RI-CLPM, prospective within-person transmission of depressive symptoms across spouses was small in all three cohorts, reaching significance under both FIML and multiple imputation in ELSA, under multiple imputation only in HRS, and not at all in CHARLS. Third, the most replicable within-person signal was the bidirectional coupling between depressive symptoms and functional limitations, with cross-spouse function-to-depression pathways detected in HRS and ELSA but not in CHARLS; sex-distinguishability tests did not reject the symmetric-dyad null in any cohort, and half-longitudinal mediation of cross-spouse depression through ADL or IADL did not survive multiplicity correction. These findings together identify the depression–disability cycle, rather than direct mood transmission, as the most tractable within-couple target for clinical intervention.

Where the present study departs from much of the prior literature on dyadic depressive concordance (Han et al., 2023; Hoppmann, Gerstorf, & Hibbert, 2011; Lu & Shelley, 2019; Meyler et al., 2007) is not in confirming the existence of concordance but in interrogating its causal interpretation. The Hamaker random-intercept specification (Hamaker et al., 2015; Mund et al., 2024) offers a more conservative test than traditional APIM. In CHARLS, once stable couple-level differences were accounted for, the depressive symptoms of one spouse at one wave were not distinguishably associated with subsequent within-person changes in the other spouse’s symptoms; in ELSA, a modest within-person cross-spouse coupling persisted; and in HRS, this coupling reached significance only in the multiple imputation-pooled estimates. This pattern is consistent with methodological work showing that traditional cross-lagged models routinely overstate interpersonal influence by absorbing trait-level similarity into the lagged paths (Berry & Willoughby, 2017; Lucas, 2023; Orth, Clark, Donnellan, & Robins, 2021). It also qualifies the closest CHARLS precedent (Liu et al., 2023): the gender-asymmetric partner effects reported in that two-wave APIM were not detected once between-couple stability was partialled out, suggesting that asymmetries observed in conventional APIM may reflect uncontrolled trait-level similarity rather than directional influence.

These results extend Lu and Shelley’s (2019) ‘shared resource’ hypothesis. Rather than depressing each other in real time, older couples may have come to share depressive states over years and decades, through assortative mating on temperament and socioeconomic position (Horwitz et al., 2023) and through cumulative joint exposure to financial, residential, and health-related stressors. What APIM captured as a ‘partner effect’ is, in the most plausible reading, the cumulative effect of these long-shared circumstances rather than a contemporaneous transfer of mood between two people. This reframing does not deny the existence of dyadic depression: concordance in our data is real and substantial in every cohort. It does, however, locate the origin of that concordance in the couple’s shared history rather than in real-time emotional contagion.

The most consistent finding at the within-person level concerns the reciprocal coupling of depressive symptoms and physical function in the two Western cohorts, reproducing at the dyadic level the depression–disability association long documented in older adults at the individual level (Penninx et al., 1998, 1999) and in recent CHARLS-based individual-level analyses (Zhou et al., 2024; Zhu et al., 2024). In our CHARLS dyadic analysis, by contrast, the same reciprocity emerged only partially: the function-to-depression direction was supported for husbands but did not reach conventional significance for wives (β = 0.025, *p* = 0.068), and the depression-to-function direction did not survive joint testing under the IADL specification. This partial attenuation may reflect both the more stringent within-person residual specification of the RI-CLPM, which removes the trait-level depression–disability covariation present in marginal-score designs, and documented spousal concordance in functional decline among married Chinese adults (Wang et al., 2021), which would inflate trait-level couple similarity in disability. More substantively novel is the cross-spouse leg of this association in HRS and ELSA: one spouse’s deteriorating functional status prospectively predicted the other spouse’s subsequent depressive symptoms, reproducing the cross-spouse stress-process pathway demonstrated in older Mexican-American couples (Monserud & Peek, 2014) and in HRS (Han, Kim, & Burr, 2021a, 2021b). This is conceptually distinct from depression ‘transmission’: one spouse’s worsening functional status plausibly raises the other’s depressive risk through tangible mechanisms theorized by the stress-process model (Pearlin et al., 1990), including rising caregiving demand and erosion of reciprocal emotional support. The corresponding cross-spouse association was not detected in CHARLS, where extended-family caregiving structures may attenuate the cross-spousal stress-process pathway compared with Western settings in which spouses are the modal caregivers (Pinquart & Sörensen, 2011).

The cross-national gradient in concordance is best read as an invitation to caution rather than a clean cultural signal. Although filial piety, multigenerational household structure, and collectivist family norms are commonly invoked to explain stronger spousal concordance in East Asian populations (Han et al., 2023; Lu & Shelley, 2019), the present design does not measure these constructs, and the same gradient is largely consistent with at least two non-cultural alternatives. Our cross-cohort MGCFA established partial metric invariance for the six common CES-D items, with the loading of the positively keyed ‘Happy’ item differing meaningfully across cohorts, while the remaining five items showed closely matched loadings; full scalar (threshold) invariance was not achieved, so quantitative comparison of latent means across cohorts is bounded even though prospective associations remain interpretable as comparable. This pattern is consistent with prior psychometric work indicating that full invariance of the CES-D often fails between Chinese and Western elderly samples, and that positively keyed items are a recurring source of noninvariance in this scale (Van de Velde, Bracke, Levecque, & Meuleman, 2010; Zhang et al., 2011). Equally important, CHARLS couples were substantially younger than HRS or ELSA couples and carried a higher baseline functional burden, so the cross-national gradient is partly confounded with cohort age and disability composition. Quantitative comparison of effect sizes across cohorts should therefore be interpreted with appropriate restraint.

Several negative findings warrant brief comment. The failure of sex-distinguishability tests to support sex-asymmetric dyadic structures stands somewhat in tension with previous within-couple analyses reporting gendered patterns of depressive concordance (Townsend, Miller, & Guo, 2001), although mixed findings are common (Ayotte, Yang, & Jones, 2010); previously reported asymmetries may partly reflect uncontrolled between-couple variance that the random-intercept specification absorbs into shared trait variance. The largely nonsignificant care-receipt moderation similarly suggests that being a care recipient is not a clean structural moderator of cross-spouse depression dynamics in these cohorts. This is consistent with population-based reappraisals indicating that informal caregiving has more heterogeneous and sometimes protective effects on caregiver health than the classical caregiver-burden literature implied (Roth, Fredman, & Haley, 2015) and with the moderation pattern reported by Han, Kim, and Burr (2021a) in HRS. The absence of formal half-longitudinal mediation through ADL or IADL despite robust bidirectional couplings in the four-variable RI-CLPM is best read methodologically; under realistic conditions of small partner-effect coefficients and age-related drift in symptom severity, the product-of-coefficients indirect effect can be vanishingly small even when each leg of the pathway is substantively meaningful (Cole & Maxwell, 2003; Maxwell, Cole, & Mitchell, 2011).

This study has several strengths. The harmonized three-cohort prospective design with extended follow-up enables direct cross-national comparison of within-couple processes over up to 16 years. The dual APIM/RI-CLPM specification explicitly separates stable between-couple similarity from prospective within-person dynamics, which conventional dyadic analyses confound. Formal indistinguishable-dyad sex-symmetry testing, multiple-imputation triangulation of FIML estimates, and pre-registered Holm and false discovery rate adjustment within hypothesis families further strengthen the inferences.

Several limitations should also be noted. First, full scalar measurement invariance of the CES-D across cohorts was not achieved, although partial metric invariance was established by MGCFA on the six common items, with the positively keyed ‘Happy’ loading freely estimated; quantitative comparison of latent means across cohorts therefore remains bounded. Second, we did not perform a formal item-level test of cross-gender measurement invariance of the CES-D, although gender is addressed through sex-stratified estimation and formal sex-distinguishability testing of all key structural paths (Supplementary Table S7). Third, differential attrition over the follow-up window was substantial, particularly in HRS and ELSA, where baseline depression and IADL limitations were significant predictors of final-wave dropout in several cohort-by-spouse models (Supplementary Table S9 Panel B); this was addressed through FIML and multiple-imputation triangulation but cannot be fully eliminated. Fourth, cultural and household-structure constructs were not directly measured, so cross-cohort comparisons cannot disentangle cultural mechanisms from other sources of between-cohort variation. Fifth, the Cole–Maxwell half-longitudinal mediation framework cannot formally test the alternative temporal ordering (functional limitations → own depression → partner depression) because reversing the predictor and mediator roles would require a different identification strategy, although the four-variable RI-CLPM jointly estimates depression-to-function and function-to-depression paths within a single model as partial converging evidence on directionality. Finally, residual confounding by unmeasured stable couple-level characteristics cannot be ruled out. As an observational cohort study, our findings should therefore be interpreted as prospective associations rather than as direct causal effects.

Late-life depressive concordance among married couples is robust and replicable across three large nationally representative aging cohorts, yet our findings indicate that it is shaped primarily by stable between-couple similarity rather than by dynamic within-person transmission. The most replicable within-person signal is the bidirectional coupling between depressive symptoms and functional limitations, observed both within and across spouses in the two Western cohorts. Clinically, these findings imply that combining depression management with functional rehabilitation and structured caregiver support represents a more coherent strategy for older couples than depression management alone, and that couple-based and family-oriented interventions for late-life depression (Belle et al., 2006; Stahl et al., 2016) offer a natural delivery framework for such combined care.

## Supporting information

10.1017/S0033291726105042.sm001 Miao et al. supplementary material Miao et al. supplementary material

## Data Availability

This study used publicly archived secondary data. CHARLS data are available from the Peking University CHARLS data portal (http://charls.pku.edu.cn) subject to a data-use agreement; HRS data from the University of Michigan Health and Retirement Study (https://hrs.isr.umich.edu) under their conditions of use; and ELSA data from the UK Data Service (https://www.elsa-project.ac.uk). Analysis code and processed datasets are available from the corresponding author upon reasonable request.

## Supplementary material

The supplementary material for this article can be found at http://doi.org/10.1017/S0033291726105042.

## Use of artificial intelligence

In line with the Cambridge University Press Artificial Intelligence Contributions to Research Content Policy, the authors disclose that generative AI tools were used in a limited and clearly defined role during the preparation of this manuscript. AI assistance was restricted to language polishing of author-drafted text, copyediting, and formatting checks against the journal’s submission requirements. AI was not used for study design, data collection, statistical analysis, interpretation of results, generation of references, or production of any scientific content. All authors verified the AI-assisted edits, take full responsibility for the accuracy, integrity, and originality of the manuscript, and confirm that no AI-generated material has been presented as their own without appropriate review. AI is not listed as an author.

## Long descriptions

### Table 1. Long description

The table is organized into three main cohort columns: C H A R L S (n = 3,532), H R S (n = 2,332), and E L S A (n = 1,895). Each cohort is subdivided into Husbands, Wives, and a p-value column comparing the two.

Key data points include:

* Age: Mean age is lowest in C H A R L S (Husbands 61.3, Wives 59.3) and highest in H R S (Husbands 67.0, Wives 64.0). All cohorts show significant age differences between spouses (p < 0.001).

* Education: C H A R L S has the highest proportion of low education (Husbands 85.8%, Wives 93.6%), while H R S has the highest proportion of high education (Husbands 31.6%, Wives 24.5%).

* C E S-D Sum Scores: C H A R L S (0-30 scale) shows baseline scores of 7.1 for husbands and 9.2 for wives. H R S and E L S A (0-8 scale) show baseline scores ranging from 0.8 to 1.3. Across all waves (W 1 to W 9), wives consistently score higher than husbands (p < 0.001 in most waves).

* Baseline Health: Self-rated health is mostly ‘Good’ in C H A R L S (approx. 50%) and ‘Fair’ or ‘Good’ in H R S. E L S A participants report higher ‘Excellent’ and ‘Very good’ health (approx. 49% combined).

* Spousal Care: In C H A R L S, 8.6% of husbands and 11.2% of wives receive care from a spouse. In E L S A, these figures are 12.2% and 20.2% respectively. H R S reports the lowest spousal care at approx. 2%.

* Functional Limitations: Baseline A D L and I A D L scores are generally low across all cohorts, with wives typically showing slightly higher mean scores than husbands.

Navigate back to Table 1..

### Table 2. Long description

The table is organized into ten columns: Transition, Outcome (M or F), n (sample size), Actor beta (S E), p F D R, Partner beta (S E), p F D R, k, 95% C I for k, and Pattern.

1. C H A R L S Cohort (2011 to 2020):

- Transitions from W 1 to W 4 consistently show a Mixed pattern for both male and female outcomes, with Actor beta values ranging from 0.354 to 0.444 and Partner beta values from 0.061 to 0.107 (all p < 0.001).

- In the W 4 to W 5 transition, the male outcome shifts to Actor-only (Partner beta 0.036, p = 0.061), while the female outcome remains Mixed.

2. H R S Cohort (2006 to 2020):

- Predominantly Actor-only patterns for both genders across most transitions.

- Exceptions include W 1 to W 2 (Female), W 2 to W 3 (Male and Female), and W 6 to W 7 (Female), which are classified as Mixed.

- Actor beta values are generally higher than C H A R L S, ranging from 0.367 to 0.575.

3. E L S A Cohort (2002 to 2018):

- Shows a high frequency of Actor-only patterns, particularly in earlier waves (W 1 to W 3) and later waves (W 6 to W 8).

- Mixed patterns appear sporadically, such as in W 3 to W 4 for both genders and W 4 to W 5 for females.

- Sample sizes (n) decrease over time from 1,471 in W 1 to W 2 down to 566 in W 8 to W 9.

Footnotes indicate that beta values are unstandardized regression coefficients on a standardized depression metric, and p F D R values are Benjamini-Hochberg adjusted.

Navigate back to Table 2..

### Figure 1. Long description

A multi-panel forest plot with three sections. Each section has an x-axis ranging from minus 0.05 to 0.25 labeled Unstandardised estimate (95% C I). Blue lines represent Autoregressive paths and orange lines represent Cross-lagged paths.

Top Panel: C H A R L S (China)

* Male A R (c M yields c M): beta equals 0.077, p less than 0.001.

* Female A R (c F yields c F): beta equals 0.085, p less than 0.001.

* Female yields Male (c F yields c M): beta equals 0.022, p equals 0.065.

* Male yields Female (c M yields c F): beta equals 0.007, p equals 0.645.

Middle Panel: H R S (United States)

* Male A R (c M yields c M): beta equals 0.162, p less than 0.001.

* Female A R (c F yields c F): beta equals 0.132, p less than 0.001.

* Female yields Male (c F yields c M): beta equals 0.014, p equals 0.292.

* Male yields Female (c M yields c F): beta equals 0.028, p equals 0.151.

Bottom Panel: E L S A (England)

* Male A R (c M yields c M): beta equals 0.193, p less than 0.001.

* Female A R (c F yields c F): beta equals 0.153, p less than 0.001.

* Female yields Male (c F yields c M): beta equals 0.026, p equals 0.030.

* Male yields Female (c M yields c F): beta equals 0.064, p less than 0.001.

In all cohorts, autoregressive paths (blue) are significantly higher and further to the right of the zero line than cross-lagged paths (orange).

Navigate back to Figure 1..

### Table 3. Long description

The table presents time-invariant within-person cross-construct paths for three cohorts.

1. C H A R L S Cohort:

- Male depression from Male A D L: Beta 0.060 (S E 0.021), p 0.004, significant.

- Male A D L from Male depression: Beta 0.073 (S E 0.025), p 0.004, significant.

- Female A D L from Female depression: Beta 0.042 (S E 0.020), p 0.037, significant.

- Other paths (spouse effects and female depression from female A D L) are non-significant at p < 0.05.

2. H R S Cohort:

- Male depression from Male A D L: Beta 0.086 (S E 0.015), p < 0.001, highly significant.

- Male depression from Female A D L: Beta 0.043 (S E 0.012), p < 0.001, highly significant.

- Male A D L from Male depression: Beta 0.104 (S E 0.030), p < 0.001, highly significant.

- Female depression from Male A D L: Beta 0.057 (S E 0.011), p < 0.001, highly significant.

- Female depression from Female A D L: Beta 0.083 (S E 0.015), p < 0.001, highly significant.

- Female A D L from Male depression: Beta 0.057 (S E 0.028), p 0.045, significant.

- Female A D L from Female depression: Beta 0.065 (S E 0.022), p 0.003, significant.

3. E L S A Cohort:

- Male depression from Male A D L: Beta 0.100 (S E 0.017), p < 0.001, highly significant.

- Male depression from Female A D L: Beta 0.043 (S E 0.017), p 0.014, significant.

- Male A D L from Male depression: Beta 0.115 (S E 0.021), p < 0.001, highly significant.

- Male A D L from Female depression: Beta 0.031 (S E 0.014), p 0.026, significant.

- Female depression from Male A D L: Beta 0.049 (S E 0.018), p 0.007, significant.

- Female depression from Female A D L: Beta 0.049 (S E 0.021), p 0.021, significant.

- Female A D L from Female depression: Beta 0.056 (S E 0.016), p < 0.001, highly significant.

Note: A D L stands for Activities of Daily Living. Significance levels are indicated by asterisks where * p < 0.05, ** p < 0.01, and *** p < 0.001.

Navigate back to Table 3..

### Figure 2. Long description

A multi-panel forest plot displays standardized estimates with 95 percent confidence intervals across three cohorts: C H A R L S (China) at the top, H R S (United States) in the middle, and E L S A (England) at the bottom.

Each panel is divided into two sections: A D L arrow Depression and Depression arrow A D L. The x-axis represents the unstandardized within-person estimate. Blue markers represent Actor (own) effects and orange markers represent Partner (spouse) effects. Solid markers indicate statistical significance at p less than 0.05, while open markers are non-significant.

* In the C H A R L S panel, most estimates hover near the zero line, with Husband Depression arrow Husband A D L and Wife A D L arrow Wife Depression showing significant positive beta values.

* In the H R S panel, all eight paths show positive estimates. Seven of the eight paths are statistically significant, with beta values ranging from 0.043 to 0.086, indicating strong cross-construct links for both actors and partners.

* In the E L S A panel, seven of the eight paths are significant and positive. The strongest effect is Husband Depression arrow Husband A D L with a beta of 0.100. The only non-significant path is Wife A D L arrow Husband Depression.

Vertical reference lines are set at 0.0 to indicate the null effect.

Navigate back to Figure 2..

## References

[r1] Andresen, E. M., Malmgren, J. A., Carter, W. B., & Patrick, D. L. (1994). Screening for depression in well older adults: Evaluation of a short form of the CES-D. American Journal of Preventive Medicine, 10(2), 77–84. 10.1016/S0749-3797(18)30622-6 8037935

[r2] Ayotte, B. J., Yang, F. M., & Jones, R. N. (2010). Physical health and depression: A dyadic study of chronic health conditions and depressive symptomatology in older adult couples. The Journals of Gerontology, Series B: Psychological Sciences and Social Sciences, 65(4), 438–448. 10.1093/geronb/gbq033 20498455 PMC2883871

[r3] Belle, S. H., Burgio, L., Burns, R., Coon, D., Czaja, S. J., Gallagher-Thompson, D., … Zhang, S. (2006). Enhancing the quality of life of dementia caregivers from different ethnic or racial groups: A randomized, controlled trial. Annals of Internal Medicine, 145(10), 727–738. 10.7326/0003-4819-145-10-200611210-00005 17116917 PMC2585490

[r4] Benjamini, Y., & Hochberg, Y. (1995). Controlling the false discovery rate: A practical and powerful approach to multiple testing. Journal of the Royal Statistical Society: Series B (Methodological), 57(1), 289–300. 10.1111/j.2517-6161.1995.tb02031.x

[r5] Berry, D., & Willoughby, M. T. (2017). On the practical interpretability of cross-lagged panel models: Rethinking a developmental workhorse. Child Development, 88(4), 1186–1206. 10.1111/cdev.12660 27878996

[r6] Boey, K. W. (1999). Cross-validation of a short form of the CES-D in Chinese elderly. International Journal of Geriatric Psychiatry, 14(8), 608–617. 10.1002/(SICI)1099-1166(199908)14:8<608::AID-GPS991>3.0.CO;2-Z 10489651

[r7] Cai, H., Jin, Y., Liu, R., Zhang, Q., Su, Z., Ungvari, G. S., … & Xiang, Y.-T. (2023). Global prevalence of depression in older adults: A systematic review and meta-analysis of epidemiological surveys. Asian Journal of Psychiatry, 80, 103417. 10.1016/j.ajp.2022.103417 36587492

[r8] Cheung, G. W., & Rensvold, R. B. (2002). Evaluating goodness-of-fit indexes for testing measurement invariance. Structural Equation Modeling, 9(2), 233–255. 10.1207/S15328007SEM0902_5

[r9] Cole, D. A., & Maxwell, S. E. (2003). Testing mediational models with longitudinal data: Questions and tips in the use of structural equation modeling. Journal of Abnormal Psychology, 112(4), 558–577. 10.1037/0021-843X.112.4.558 14674869

[r10] Cook, W. L., & Kenny, D. A. (2005). The actor–partner interdependence model: A model of bidirectional effects in developmental studies. International Journal of Behavioral Development, 29(2), 101–109. 10.1080/01650250444000405

[r11] GBD. (2022). Global, regional, and national burden of 12 mental disorders in 204 countries and territories, 1990-2019: A systematic analysis for the global burden of disease study 2019. The Lancet Psychiatry, 9(2), 137–150. 10.1016/S2215-0366(21)00395-3 35026139 PMC8776563

[r12] Hamaker, E. L., Kuiper, R. M., & Grasman, R. P. P. P. (2015). A critique of the cross-lagged panel model. Psychological Methods, 20(1), 102–116. 10.1037/a0038889 25822208

[r13] Han, J. W., Yang, H. W., Bae, J. B., Oh, D. J., Moon, D. G., Lim, E., … & Kim, K. W. (2023). Shared risk factors for depressive disorder among older adult couples in Korea. JAMA Network Open, 6(4), e238263. 10.1001/jamanetworkopen.2023.8263 37058304 PMC10105310

[r14] Han, S. H., Kim, K., & Burr, J. A. (2021a). Activity limitations and depressive symptoms among older couples: The moderating role of spousal care. The Journals of Gerontology, Series B: Psychological Sciences and Social Sciences, 76(2), 360–369. 10.1093/geronb/gbz161 31883010 PMC8435753

[r15] Han, S. H., Kim, K., & Burr, J. A. (2021b). Take a sad song and make it better: Spousal activity limitations, caregiving, and depressive symptoms among couples. Social Science & Medicine, 281, 114081. 10.1016/j.socscimed.2021.114081 34091231 PMC8277459

[r16] Holm, S. (1979). A simple sequentially rejective multiple test procedure. Scandinavian Journal of Statistics, 6(2), 65–70.

[r17] Hoppmann, C. A., Gerstorf, D., & Hibbert, A. (2011). Spousal associations between functional limitation and depressive symptom trajectories: Longitudinal findings from the study of asset and health dynamics among the oldest old (AHEAD). Health Psychology, 30(2), 153–162. 10.1037/a0022094 21401249 PMC3078040

[r18] Horwitz, T. B., Balbona, J. V., Paulich, K. N., & Keller, M. C. (2023). Evidence of correlations between human partners based on systematic reviews and meta-analyses of 22 traits and UK biobank analysis of 133 traits. Nature Human Behaviour, 7(9), 1568–1583. 10.1038/s41562-023-01672-z PMC10967253 37653148

[r19] Kenny, D. A., Kashy, D. A., & Cook, W. L. (2006). Dyadic data analysis. Guilford Press.

[r20] Kenny, D. A., & Ledermann, T. (2010). Detecting, measuring, and testing dyadic patterns in the actor–partner interdependence model. Journal of Family Psychology, 24(3), 359–366. 10.1037/a0019651 20545409

[r21] Kiecolt-Glaser, J. K., & Wilson, S. J. (2017). Lovesick: How couples’ relationships influence health. Annual Review of Clinical Psychology, 13(1), 421–443. 10.1146/annurev-clinpsy-032816-045111 PMC5549103 28301763

[r22] Kohout, F. J., Berkman, L. F., Evans, D. A., & Cornoni-Huntley, J. (1993). Two shorter forms of the CES-D depression symptoms index. Journal of Aging and Health, 5(2), 179–193. 10.1177/089826439300500202 10125443

[r23] Liu, H., Ma, Y., Lin, L., Sun, Z., Li, Z., & Jiang, X. (2023). Association between activities of daily living and depressive symptoms among older adults in China: Evidence from the CHARLS. Frontiers in Public Health, 11, 1249208. 10.3389/fpubh.2023.1249208 38035294 PMC10687586

[r24] Liu, J., Lou, Y., Li, L. W., Xu, H., & Zhang, Z. (2023). Dyadic effects on depressive symptoms of spouse caregivers and their care recipients: Evidence from China. Aging & Mental Health, 27(7), 1256–1265. 10.1080/13607863.2022.2087212 35694965

[r25] Lu, P., & Shelley, M. (2019). Why spouses depress each other: A cross-national study to test the shared resource hypothesis in depressive symptom concordance within older adult couples. Asian Social Work and Policy Review, 13(3), 307–319. 10.1111/aswp.12183 35677604 PMC9173758

[r26] Lucas, R. E. (2023). Why the cross-lagged panel model is almost never the right choice. Advances in Methods and Practices in Psychological Science, 6(1), 25152459231158378. 10.1177/25152459231158378

[r27] Maxwell, S. E., Cole, D. A., & Mitchell, M. A. (2011). Bias in cross-sectional analyses of longitudinal mediation: Partial and complete mediation under an autoregressive model. Multivariate Behavioral Research, 46(5), 816–841. 10.1080/00273171.2011.606716 26736047

[r28] Meyler, D., Stimpson, J. P., & Peek, M. K. (2007). Health concordance within couples: A systematic review. Social Science & Medicine, 64(11), 2297–2310. 10.1016/j.socscimed.2007.02.007 17374552

[r29] Monserud, M. A., & Peek, M. K. (2014). Functional limitations and depressive symptoms: A longitudinal analysis of older Mexican American couples. The Journals of Gerontology, Series B: Psychological Sciences and Social Sciences, 69(5), 743–762. 10.1093/geronb/gbu039 24823692 PMC4189648

[r30] Mulder, J. D., & Hamaker, E. L. (2021). Three extensions of the random intercept cross-lagged panel model. Structural Equation Modeling: A Multidisciplinary Journal, 28(4), 638–648. 10.1080/10705511.2020.1784738

[r31] Mund, M., Park, Y., & Nestler, S. (2024). Disentangling between- and within-person variation in relationship science. Journal of Marriage and Family, 86(5), 1495–1518. 10.1111/jomf.12999

[r32] Orth, U., Clark, D. A., Donnellan, M. B., & Robins, R. W. (2021). Testing prospective effects in longitudinal research: Comparing seven competing cross-lagged models. Journal of Personality and Social Psychology, 120(4), 1013–1034. 10.1037/pspp0000358 32730068 PMC7854859

[r33] Pearlin, L. I., Mullan, J. T., Semple, S. J., & Skaff, M. M. (1990). Caregiving and the stress process: An overview of concepts and their measures. The Gerontologist, 30(5), 583–594. 10.1093/geront/30.5.583 2276631

[r34] Penninx, B. W. J. H., Guralnik, J. M., Ferrucci, L., Simonsick, E. M., Deeg, D. J. H., & Wallace, R. B. (1998). Depressive symptoms and physical decline in community-dwelling older persons. JAMA, 279(21), 1720–1726. 10.1001/jama.279.21.1720 9624025

[r35] Penninx, B. W. J. H., Leveille, S., Ferrucci, L., van Eijk, J. T., & Guralnik, J. M. (1999). Exploring the effect of depression on physical disability: Longitudinal evidence from the established populations for epidemiologic studies of the elderly. American Journal of Public Health, 89(9), 1346–1352. 10.2105/AJPH.89.9.1346 10474551 PMC1508750

[r36] Pinquart, M., & Sörensen, S. (2011). Spouses, adult children, and children-in-law as caregivers of older adults: A meta-analytic comparison. Psychology and Aging, 26(1), 1–14. 10.1037/a0021863 21417538 PMC4449135

[r37] Preacher, K. J., & Hayes, A. F. (2008). Asymptotic and resampling strategies for assessing and comparing indirect effects in multiple mediator models. Behavior Research Methods, 40(3), 879–891. 10.3758/BRM.40.3.879 18697684

[r38] Rosseel, Y. (2012). Lavaan: AnRPackage for structural equation modeling. Journal of Statistical Software, 48(2), 1–36. 10.18637/jss.v048.i02

[r39] Roth, D. L., Fredman, L., & Haley, W. E. (2015). Informal caregiving and its impact on health: A reappraisal from population-based studies. The Gerontologist, 55(2), 309–319. 10.1093/geront/gnu177 26035608 PMC6584119

[r40] Satorra, A., & Bentler, P. M. (2010). Ensuring positiveness of the scaled difference chi-square test statistic. Psychometrika, 75(2), 243–248. 10.1007/s11336-009-9135-y 20640194 PMC2905175

[r41] Sonnega, A., Faul, J. D., Ofstedal, M. B., Langa, K. M., Phillips, J. W. R., & Weir, D. R. (2014). Cohort profile: The health and retirement study (HRS). International Journal of Epidemiology, 43(2), 576–585. 10.1093/ije/dyu067 24671021 PMC3997380

[r42] Stahl, S. T., Rodakowski, J., Saghafi, E. M., Park, M., Reynolds, C. F., & Dew, M. A. (2016). Systematic review of dyadic and family-oriented interventions for late-life depression. International Journal of Geriatric Psychiatry, 31(9), 963–973. 10.1002/gps.4434 26799782 PMC5166608

[r43] Steptoe, A., Breeze, E., Banks, J., & Nazroo, J. (2013). Cohort profile: The English longitudinal study of ageing. International Journal of Epidemiology, 42(6), 1640–1648. 10.1093/ije/dys168 23143611 PMC3900867

[r44] Sun, Q., Wei, Y., Xie, H., Lyu, J., Zhou, J., Li, X., … Li, S. (2025). The global, regional, and national late-life depression burden and trends from 1990 to 2021: A systematic analysis for the global burden of disease study 2021. Archives of Gerontology and Geriatrics, 131, 105758. 10.1016/j.archger.2025.105758 39874854

[r45] Sun, W., Ren, Z., Zhu, S., Cheng, S., Liu, W., Li, H. C. W., … & Song, P. (2023). Spousal concordance in adverse childhood experiences and the association with depressive symptoms in middle-aged and older adults: Findings across China, the US, and Europe. Frontiers in Public Health, 11, 1158590. 10.3389/fpubh.2023.1158590 37383257 PMC10297162

[r46] Townsend, A. L., Miller, B., & Guo, S. (2001). Depressive symptomatology in middle-aged and older married couples: A dyadic analysis. The Journals of Gerontology, Series B: Psychological Sciences and Social Sciences, 56(6), S352–S364. 10.1093/geronb/56.6.S352 11682596

[r47] van Buuren, S., & Groothuis-Oudshoorn, K. (2011). Mice: Multivariate imputation by chained equations inR. Journal of Statistical Software, 45(3), 1–67. 10.18637/jss.v045.i03

[r48] Van de Velde, S., Bracke, P., Levecque, K., & Meuleman, B. (2010). Gender differences in depression in 25 European countries after eliminating measurement bias in the CES-D 8. Social Science Research, 39(3), 396–404. 10.1016/j.ssresearch.2010.01.002

[r49] Wang, J., Wang, Q., Hou, X.-Y., Chen, S., Guo, Z., Du, W., & Fan, L. (2021). Spousal concordance in the development of functional limitations among married adults in China. JAMA Network Open, 4(9), e2125577. 10.1001/jamanetworkopen.2021.25577 34581797 PMC8479583

[r50] Zhang, B., Fokkema, M., Cuijpers, P., Li, J., Smits, N., & Beekman, A. (2011). Measurement invariance of the Center for Epidemiological Studies Depression Scale (CES-D) among Chinese and Dutch elderly. BMC Medical Research Methodology, 11(1), 74. 10.1186/1471-2288-11-74 21595982 PMC3123656

[r51] Zhao, Y., Hu, Y., Smith, J. P., Strauss, J., & Yang, G. (2014). Cohort profile: The China health and retirement longitudinal study (CHARLS). International Journal of Epidemiology, 43(1), 61–68. 10.1093/ije/dys203 23243115 PMC3937970

[r52] Zhou, L., Wang, W., & Ma, X. (2024). The bidirectional association between the disability in activities of daily living and depression: A longitudinal study in Chinese middle-aged and older adults. BMC Public Health, 24(1), 1884. 10.1186/s12889-024-19421-w 39010036 PMC11247890

[r53] Zhu, X., Wang, Y., Luo, Y., Ding, R., Shi, Z., & He, P. (2024). Bidirectional, longitudinal associations between depressive symptoms and IADL/ADL disability in older adults in China: A national cohort study. BMC Geriatrics, 24(1), 659. 10.1186/s12877-024-05248-y 39107705 PMC11301930

