# Supplementary material for: Spousal concordance and interdependence in depression among older adults: A cross-cultural longitudinal study in China, the United States, and England
Source: Psychol Med. 2026 Jul 29;56:e241. doi: 10.1017/S0033291726105042 (PMC13439253; doi:10.1017/S0033291726105042)
Supplement: Supplementary file 1 [file S0033291726105042sup001.docx]

**Supplementary Materials**

**Table S1.** Within-couple similarity of standardized depressive symptoms by wave in each cohort.

**Table S2.** Multiple-imputation-pooled APIM partner-effect estimates with fraction of missing information, plus z-versus-binary-cutoff CES-D comparability summary, by cohort.

**Table S3.** Three-wave longitudinal mediation: a priori primary pathways with indirect-effect estimates, 95% bootstrap CIs, and Holm-adjusted p-values, by cohort.

**Table S4.** Care-receipt moderation of the partner effect: joint Wald F test of the two partner × care-category interactions per APIM transition, by cohort.

**Table S5.** Bivariate Random-Intercept Cross-Lagged Panel Model: model-fit indices, time-invariant within-person cross-lagged paths, and between-person random-intercept structure, by cohort.

**Table S6.** Bivariate Random-Intercept Cross-Lagged Panel Model: multiple-imputation-pooled within-person cross-lagged paths, by cohort.

**Table S7.** Sex-distinguishability tests at each wave transition: joint Wald test of role × actor and role × partner interactions in long-format dyadic regressions, by cohort.

**Table S8.** Reporting-bias robustness analyses: four-variable Random-Intercept Cross-Lagged Panel Model with IADL functional status, Satorra–Bentler scaled difference test, and bivariate-versus-four-variable attenuation diagnostic, by cohort.

**Table S9.** Missing-data mechanism: Little’s MCAR test and logistic-regression prediction of final-wave dropout, by cohort.

**Table S10.** Cross-cohort multi-group confirmatory factor analysis (MGCFA) of the six common CES-D items: model-fit indices under both CHARLS-binarisation thresholds.

**Table S11.** Standardised factor loadings for the six common CES-D items in the configural MGCFA, by cohort.

**Figure S1.** Wave-by-wave evolution of CES-D depressive-symptom scores by gender in each cohort.

**Figure S2.** Wave-by-wave evolution of functional limitations (ADL, range 0–6; IADL, range 0–5) by gender in each cohort.

**Figure S3.** Composition of spousal-care status across survey waves, by gender and cohort.

**Figure S4.** Within-couple similarity of depressive symptoms over time, by cohort.

**Figure S5.** Wave-specific Actor–Partner Interdependence Model cross-lagged coefficients, comparing complete-case (listwise) and multiple-imputation pooled estimates, by cohort.

**Figure S6.** Confounding by stable between-couple similarity: traditional Cross-Lagged Panel Model estimates versus Random-Intercept Cross-Lagged Panel Model within-person estimates, by cohort.

**Table S1. Within-couple similarity of standardized depressive symptoms by wave in each cohort.**

| **Cohort · wave** | **Year** | **Dyads (*n*)** | **Pearson *r*** | **95% CI** | **ICC** | **95% CI** |
| --- | --- | --- | --- | --- | --- | --- |
| ***CHARLS*** | | | | | | |
| W1 | 2011 | 3,201 | 0.432 | (0.400, 0.465) | 0.386 | (0.370, 0.403) |
| W2 | 2013 | 3,065 | 0.380 | (0.343, 0.412) | 0.339 | (0.323, 0.355) |
| W3 | 2015 | 3,284 | 0.343 | (0.307, 0.377) | 0.301 | (0.286, 0.315) |
| W4 | 2018 | 2,838 | 0.355 | (0.321, 0.390) | 0.320 | (0.304, 0.336) |
| W5 | 2020 | 2,167 | 0.418 | (0.379, 0.456) | 0.380 | (0.363, 0.397) |
| ***HRS*** | | | | | | |
| W1 | 2006 | 2,194 | 0.197 | (0.146, 0.248) | 0.189 | (0.177, 0.203) |
| W2 | 2008 | 2,219 | 0.139 | (0.084, 0.194) | 0.133 | (0.124, 0.143) |
| W3 | 2010 | 2,175 | 0.204 | (0.146, 0.260) | 0.195 | (0.182, 0.208) |
| W4 | 2012 | 1,992 | 0.153 | (0.101, 0.210) | 0.152 | (0.141, 0.164) |
| W5 | 2014 | 1,692 | 0.188 | (0.128, 0.249) | 0.185 | (0.171, 0.199) |
| W6 | 2016 | 1,370 | 0.153 | (0.085, 0.221) | 0.151 | (0.138, 0.165) |
| W7 | 2018 | 1,067 | 0.158 | (0.073, 0.246) | 0.155 | (0.140, 0.171) |
| W8 | 2020 | 874 | 0.146 | (0.062, 0.231) | 0.130 | (0.116, 0.146) |
| ***ELSA*** | | | | | | |
| W1 | 2002 | 1,849 | 0.248 | (0.195, 0.300) | 0.223 | (0.208, 0.240) |
| W2 | 2004 | 1,792 | 0.254 | (0.199, 0.314) | 0.229 | (0.213, 0.246) |
| W3 | 2006 | 1,752 | 0.234 | (0.178, 0.290) | 0.209 | (0.194, 0.225) |
| W4 | 2008 | 1,478 | 0.269 | (0.205, 0.330) | 0.242 | (0.224, 0.261) |
| W5 | 2010 | 1,344 | 0.282 | (0.214, 0.352) | 0.272 | (0.251, 0.293) |
| W6 | 2012 | 1,189 | 0.279 | (0.204, 0.349) | 0.245 | (0.224, 0.266) |
| W7 | 2014 | 1,006 | 0.278 | (0.205, 0.351) | 0.254 | (0.231, 0.278) |
| W8 | 2016 | 854 | 0.276 | (0.205, 0.353) | 0.233 | (0.210, 0.258) |
| W9 | 2018 | 698 | 0.288 | (0.197, 0.382) | 0.240 | (0.214, 0.268) |

***Note.*** Pearson *r* is the cross-spouse correlation with a 1,000-resample bootstrap 95% CI; ICC is the two-way intraclass correlation estimated from a random-intercept linear mixed model with member nested in couple. All correlations are statistically distinguishable from zero (*p* < 0.001).

**Table S2. Multiple-imputation-pooled APIM partner-effect estimates with fraction of missing information, plus z-versus-binary-cutoff CES-D comparability summary, by cohort.**

***Panel A. Rubin-rule-pooled APIM partner-effect estimates (m = 20 imputations).***

| **Transition** | **Outcome** | **Partner β (SE) MI** | ***p* FDR** | ***p* Holm** | **FMI** | ***k* MI** | **Pattern (MI)** |
| --- | --- | --- | --- | --- | --- | --- | --- |
| ***CHARLS*** | | | | | | | |
| W1→W2 (2011–13) | M | 0.051 (0.015) | 0.001 | 0.003 | 0.141 | 0.144 | mixed |
| W1→W2 (2011–13) | F | 0.048 (0.018) | 0.012 | 0.028 | 0.073 | 0.134 | mixed |
| W2→W3 (2013–15) | M | 0.075 (0.017) | < 0.001 | < 0.001 | 0.098 | 0.174 | mixed |
| W2→W3 (2013–15) | F | 0.071 (0.022) | 0.002 | 0.005 | 0.129 | 0.159 | mixed |
| W3→W4 (2015–18) | M | 0.069 (0.018) | < 0.001 | < 0.001 | 0.193 | 0.166 | mixed |
| W3→W4 (2015–18) | F | 0.090 (0.024) | < 0.001 | < 0.001 | 0.124 | 0.225 | mixed |
| W4→W5 (2018–20) | M | 0.023 (0.022) | 0.172 | 0.172 | 0.279 | 0.060 | actor-only |
| W4→W5 (2018–20) | F | 0.047 (0.024) | 0.030 | 0.053 | 0.283 | 0.108 | mixed |
| ***HRS*** | | | | | | | |
| W1→W2 (06–08) | M | 0.003 (0.017) | .909 | 1.000 | 0.031 | 0.006 | actor-only |
| W1→W2 (06–08) | F | 0.060 (0.023) | .028 | .100 | 0.045 | 0.151 | mixed |
| W2→W3 (08–10) | M | 0.047 (0.016) | .019 | .050 | 0.054 | 0.102 | mixed |
| W2→W3 (08–10) | F | 0.079 (0.022) | .005 | .005 | 0.045 | 0.212 | mixed |
| W3→W4 (10–12) | M | 0.019 (0.018) | .438 | 1.000 | 0.073 | 0.041 | actor-only |
| W3→W4 (10–12) | F | −0.007 (0.024) | .897 | 1.000 | 0.105 | −0.018 | actor-only |
| W4→W5 (12–14) | M | 0.057 (0.020) | .019 | .059 | 0.298 | 0.116 | mixed |
| W4→W5 (12–14) | F | 0.058 (0.024) | .035 | .135 | 0.101 | 0.135 | mixed |
| W5→W6 (14–16) | M | 0.025 (0.021) | .353 | 1.000 | 0.392 | 0.054 | actor-only |
| W5→W6 (14–16) | F | 0.043 (0.026) | .209 | .835 | 0.289 | 0.110 | actor-only |
| W6→W7 (16–18) | M | −0.017 (0.020) | .509 | 1.000 | 0.308 | −0.035 | actor-only |
| W6→W7 (16–18) | F | 0.087 (0.031) | .019 | .060 | 0.483 | 0.192 | mixed |
| W7→W8 (18–20) | M | 0.030 (0.024) | .353 | 1.000 | 0.528 | 0.061 | actor-only |
| W7→W8 (18–20) | F | 0.003 (0.027) | .909 | 1.000 | 0.305 | 0.006 | actor-only |
| ***ELSA*** | | | | | | | |
| W1→W2 (02–04) | M | 0.010 (0.018) | 0.652 | 1.000 | 0.019 | 0.026 | actor-only |
| W1→W2 (02–04) | F | 0.030 (0.027) | 0.375 | 1.000 | 0.032 | 0.086 | actor-only |
| W2→W3 (04–06) | M | −0.009 (0.018) | 0.652 | 1.000 | 0.073 | −0.023 | actor-only |
| W2→W3 (04–06) | F | 0.034 (0.026) | 0.312 | 1.000 | 0.056 | 0.083 | actor-only |
| W3→W4 (06–08) | M | 0.057 (0.021) | 0.015 | 0.063 | 0.270 | 0.132 | mixed |
| W3→W4 (06–08) | F | 0.092 (0.030) | 0.008 | 0.028 | 0.160 | 0.235 | mixed |
| W4→W5 (08–10) | M | 0.024 (0.022) | 0.375 | 1.000 | 0.295 | 0.054 | actor-only |
| W4→W5 (08–10) | F | 0.131 (0.030) | < 0.001 | < 0.001 | 0.193 | 0.359 | mixed |
| W5→W6 (10–12) | M | 0.070 (0.020) | 0.003 | 0.010 | 0.335 | 0.215 | mixed |
| W5→W6 (10–12) | F | 0.052 (0.033) | 0.207 | 0.931 | 0.442 | 0.116 | actor-only |
| W6→W7 (12–14) | M | 0.019 (0.023) | 0.506 | 1.000 | 0.400 | 0.047 | actor-only |
| W6→W7 (12–14) | F | 0.088 (0.038) | 0.045 | 0.203 | 0.514 | 0.211 | mixed |
| W7→W8 (14–16) | M | 0.059 (0.022) | 0.017 | 0.074 | 0.403 | 0.154 | mixed |
| W7→W8 (14–16) | F | 0.010 (0.034) | 0.778 | 1.000 | 0.388 | 0.021 | actor-only |
| W8→W9 (16–18) | M | 0.073 (0.020) | 0.002 | 0.006 | 0.362 | 0.171 | mixed |
| W8→W9 (16–18) | F | 0.159 (0.038) | < 0.001 | < 0.001 | 0.444 | 0.417 | mixed |

***Panel B. z-Score-APIM versus binary-cutoff-APIM comparability (Benjamini–Hochberg-adjusted partner-effect agreement).***

| **Cohort** | **Binary cut-off** | **z-score vs LPM agreement** | **z-score vs logistic agreement** |
| --- | --- | --- | --- |
| CHARLS | CES-D-10 ≥ 10 | 7/8 (87.5%) | 7/8 (87.5%) |
| HRS | CES-D-8 ≥ 3 | 11/14 (78.6%) | 11/14 (78.6%) |
| ELSA | CES-D-8 ≥ 3 | 16/16 (100.0%) | 15/16 (93.8%) |

***Note.*** Panel A reports APIM partner effects pooled across m = 20 multiple-imputation datasets via Rubin’s rules. FMI = fraction of missing information. Pattern (MI) is the dyadic-pattern classification using the pooled point estimates and SEs. Panel B reports cross-scale comparability between the within-cohort z-standardized APIM (main analysis) and a binary-cutoff APIM that re-estimates each cell at CES-D-10 ≥ 10 or CES-D-8 ≥ 3 using a linear probability model (LPM, HC3 errors) and a logistic model. Agreement is the proportion of partner-effect significance verdicts that match the z-score-APIM verdict after Benjamini–Hochberg adjustment.

**Table S3. Three-wave longitudinal mediation: a priori primary pathways with indirect-effect estimates, 95% bootstrap CIs, and Holm-adjusted p-values, by cohort.**

| **Period** | **Pathway** | ***n*** | ***a* path** | ***b* path** | **Indirect (*ab*)** | **95% bootstrap CI** | ***p* boot** | ***p* Holm** |
| --- | --- | --- | --- | --- | --- | --- | --- | --- |
| ***CHARLS*** | | | | | | | | |
| W1–W2–W3 | M_dep → M_ADL → F_dep | 2,846 | 0.048 | 0.049 | 0.0023 | (0.0001, 0.0062) | 0.070 | 0.560 |
| W1–W2–W3 | F_dep → F_ADL → M_dep | 2,858 | 0.093 | 0.028 | 0.0026 | (−0.0006, 0.0070) | 0.198 | 0.828 |
| W1–W2–W3 | M_dep → M_IADL → F_dep | 2,801 | 0.039 | 0.041 | 0.0016 | (0.0000, 0.0066) | 0.144 | 0.828 |
| W1–W2–W3 | F_dep → F_IADL → M_dep | 2,819 | 0.053 | 0.075 | 0.0040 | (0.0009, 0.0093) | 0.014 | 0.140 |
| W2–W3–W4 | M_dep → M_ADL → F_dep | 2,550 | 0.042 | 0.039 | 0.0016 | (−0.0001, 0.0053) | 0.138 | 0.828 |
| W2–W3–W4 | F_dep → F_ADL → M_dep | 2,466 | 0.102 | 0.067 | 0.0068 | (0.0021, 0.0139) | 0.008 | 0.096 |
| W2–W3–W4 | M_dep → M_IADL → F_dep | 2,521 | 0.011 | 0.076 | 0.0008 | (−0.0022, 0.0047) | 0.606 | 0.828 |
| W2–W3–W4 | F_dep → F_IADL → M_dep | 2,429 | 0.136 | 0.038 | 0.0051 | (−0.0008, 0.0128) | 0.096 | 0.672 |
| W3–W4–W5 | M_dep → M_ADL → F_dep | 2,225 | 0.037 | 0.039 | 0.0014 | (−0.0001, 0.0053) | 0.178 | 0.828 |
| W3–W4–W5 | F_dep → F_ADL → M_dep | 2,271 | 0.049 | 0.058 | 0.0029 | (0.0004, 0.0078) | 0.040 | 0.360 |
| W3–W4–W5 | M_dep → M_IADL → F_dep | 2,225 | 0.039 | 0.037 | 0.0014 | (−0.0001, 0.0055) | 0.168 | 0.828 |
| W3–W4–W5 | F_dep → F_IADL → M_dep | 2,271 | 0.100 | 0.052 | 0.0052 | (0.0014, 0.0119) | 0.008 | 0.096 |
| ***HRS (selected periods)*** | | | | | | | | |
| W1–2–3 | M_dep → M_ADL → F_dep | 2,104 | 0.041 | 0.001 | 0.0000 | (−0.0019, 0.0040) | 0.904 | 1.000 |
| W1–2–3 | F_dep → F_ADL → M_dep | 2,088 | 0.022 | 0.047 | 0.0010 | (−0.0006, 0.0059) | 0.472 | 1.000 |
| W1–2–3 | M_dep → M_IADL → F_dep | 2,104 | 0.053 | 0.003 | 0.0001 | (−0.0025, 0.0035) | 0.902 | 1.000 |
| W1–2–3 | F_dep → F_IADL → M_dep | 2,088 | 0.044 | 0.029 | 0.0013 | (−0.0009, 0.0078) | 0.388 | 1.000 |
| W2–3–4 | M_dep → M_ADL → F_dep | 2,035 | 0.079 | 0.056 | 0.0044 | (0.0005, 0.0125) | 0.068 | 1.000 |
| W2–3–4 | F_dep → F_ADL → M_dep | 1,969 | 0.033 | 0.030 | 0.0010 | (−0.0009, 0.0076) | 0.580 | 1.000 |
| W2–3–4 | M_dep → M_IADL → F_dep | 2,035 | 0.086 | 0.073 | 0.0062 | (0.0006, 0.0178) | 0.040 | 0.960 |
| W2–3–4 | F_dep → F_IADL → M_dep | 1,968 | −0.024 | 0.050 | −0.0012 | (−0.0070, 0.0005) | 0.438 | 1.000 |
| W3–4–5 | M_dep → M_ADL → F_dep | 1,777 | 0.021 | 0.022 | 0.0005 | (−0.0009, 0.0053) | 0.662 | 1.000 |
| W3–4–5 | F_dep → F_ADL → M_dep | 1,679 | 0.012 | 0.032 | 0.0004 | (−0.0010, 0.0056) | 0.764 | 1.000 |
| W3–4–5 | M_dep → M_IADL → F_dep | 1,776 | −0.003 | −0.009 | 0.0000 | (−0.0018, 0.0025) | 0.914 | 1.000 |
| W3–4–5 | F_dep → F_IADL → M_dep | 1,679 | 0.033 | 0.035 | 0.0012 | (−0.0004, 0.0071) | 0.350 | 1.000 |
| W4–5–6 | M_dep → M_ADL → F_dep | 1,497 | 0.058 | 0.101 | 0.0059 | (0.0006, 0.0171) | 0.072 | 1.000 |
| W4–5–6 | F_dep → F_ADL → M_dep | 1,413 | −0.027 | −0.009 | 0.0003 | (−0.0023, 0.0058) | 0.910 | 1.000 |
| W4–5–6 | M_dep → M_IADL → F_dep | 1,497 | 0.061 | 0.073 | 0.0045 | (0.0002, 0.0149) | 0.090 | 1.000 |
| W4–5–6 | F_dep → F_IADL → M_dep | 1,411 | 0.009 | 0.032 | 0.0003 | (−0.0017, 0.0056) | 0.896 | 1.000 |
| W5–6–7 | M_dep → M_ADL → F_dep | 1,191 | −0.072 | 0.043 | −0.0031 | (−0.0142, 0.0017) | 0.332 | 1.000 |
| W5–6–7 | F_dep → F_ADL → M_dep | 1,120 | −0.033 | 0.027 | −0.0009 | (−0.0071, 0.0011) | 0.600 | 1.000 |
| W5–6–7 | M_dep → M_IADL → F_dep | 1,191 | −0.064 | 0.020 | −0.0013 | (−0.0087, 0.0029) | 0.568 | 1.000 |
| W5–6–7 | F_dep → F_IADL → M_dep | 1,119 | 0.011 | 0.082 | 0.0009 | (−0.0031, 0.0085) | 0.764 | 1.000 |
| W6–7–8 | M_dep → M_ADL → F_dep | 977 | −0.007 | 0.058 | −0.0004 | (−0.0058, 0.0043) | 0.860 | 1.000 |
| W6–7–8 | F_dep → F_ADL → M_dep | 902 | 0.027 | 0.098 | 0.0027 | (−0.0020, 0.0123) | 0.376 | 1.000 |
| W6–7–8 | M_dep → M_IADL → F_dep | 977 | 0.018 | 0.103 | 0.0019 | (−0.0056, 0.0153) | 0.652 | 1.000 |
| W6–7–8 | F_dep → F_IADL → M_dep | 901 | 0.018 | 0.069 | 0.0012 | (−0.0021, 0.0081) | 0.562 | 1.000 |
| ***ELSA (selected periods)*** | | | | | | | | |
| W1–W2–W3 | M_dep → M_ADL → F_dep | 1,415 | 0.037 | 0.013 | 0.0005 | (−0.0022, 0.0067) | 0.766 | 1.000 |
| W1–W2–W3 | F_dep → F_ADL → M_dep | 1,421 | 0.056 | −0.017 | −0.0009 | (−0.0058, 0.0023) | 0.586 | 1.000 |
| W1–W2–W3 | M_dep → M_IADL → F_dep | 1,415 | 0.038 | 0.017 | 0.0006 | (−0.0013, 0.0063) | 0.648 | 1.000 |
| W1–W2–W3 | F_dep → F_IADL → M_dep | 1,421 | 0.014 | 0.023 | 0.0003 | (−0.0008, 0.0040) | 0.934 | 1.000 |
| W2–W3–W4 | M_dep → M_ADL → F_dep | 1,223 | 0.096 | 0.087 | 0.0084 | (0.0013, 0.0239) | 0.028 | 0.728 |
| W2–W3–W4 | F_dep → F_ADL → M_dep | 1,195 | 0.087 | 0.023 | 0.0020 | (−0.0039, 0.0124) | 0.566 | 1.000 |
| W2–W3–W4 | M_dep → M_IADL → F_dep | 1,223 | 0.022 | 0.035 | 0.0008 | (−0.0008, 0.0066) | 0.528 | 1.000 |
| W2–W3–W4 | F_dep → F_IADL → M_dep | 1,195 | 0.053 | 0.052 | 0.0027 | (−0.0011, 0.0111) | 0.266 | 1.000 |
| W3–W4–W5 | M_dep → M_ADL → F_dep | 1,080 | 0.033 | 0.034 | 0.0011 | (−0.0018, 0.0092) | 0.662 | 1.000 |
| W3–W4–W5 | F_dep → F_ADL → M_dep | 1,045 | 0.099 | 0.100 | 0.0100 | (0.0021, 0.0250) | 0.020 | 0.540 |
| W3–W4–W5 | M_dep → M_IADL → F_dep | 1,080 | 0.009 | 0.101 | 0.0009 | (−0.0046, 0.0087) | 0.770 | 1.000 |
| W3–W4–W5 | F_dep → F_IADL → M_dep | 1,045 | 0.028 | 0.132 | 0.0037 | (−0.0024, 0.0160) | 0.342 | 1.000 |
| W4–W5–W6 | M_dep → M_ADL → F_dep | 973 | 0.042 | 0.017 | 0.0007 | (−0.0022, 0.0077) | 0.726 | 1.000 |
| W4–W5–W6 | F_dep → F_ADL → M_dep | 960 | 0.016 | −0.021 | −0.0003 | (−0.0066, 0.0022) | 0.950 | 1.000 |
| W4–W5–W6 | M_dep → M_IADL → F_dep | 973 | 0.092 | 0.077 | 0.0071 | (−0.0026, 0.0285) | 0.278 | 1.000 |
| W4–W5–W6 | F_dep → F_IADL → M_dep | 960 | 0.001 | 0.027 | 0.0000 | (−0.0051, 0.0047) | 0.774 | 1.000 |
| W5–W6–W7 | M_dep → M_ADL → F_dep | 874 | −0.002 | 0.049 | −0.0001 | (−0.0057, 0.0034) | 0.936 | 1.000 |
| W5–W6–W7 | F_dep → F_ADL → M_dep | 851 | 0.112 | 0.111 | 0.0124 | (0.0028, 0.0292) | 0.012 | 0.336 |
| W5–W6–W7 | M_dep → M_IADL → F_dep | 874 | 0.010 | 0.084 | 0.0008 | (−0.0035, 0.0113) | 0.738 | 1.000 |
| W5–W6–W7 | F_dep → F_IADL → M_dep | 851 | 0.127 | 0.079 | 0.0100 | (−0.0011, 0.0277) | 0.170 | 1.000 |

***Note.*** Three-wave half-longitudinal mediation: predictor at wave t, mediator at wave t+1, outcome at wave t+2, adjusting for the outcome spouse’s baseline depression and both spouses’ baseline ADL, IADL, and self-rated health. Indirect (*ab*) is the standardized indirect effect with a 1,000-resample bias-corrected bootstrap 95% CI; *p* boot is the two-sided bootstrap p-value, *p* Holm is the Holm-adjusted p-value within the family of a priori primary pathways. M = male; F = female. The Holm correction was applied across the full set of pre-specified primary a priori pathways for each cohort (CHARLS, 12; HRS, 24; ELSA, 28), and only the periods with at least one |*ab*| reaching the uncorrected screening threshold (p < 0.20) are listed in the table; therefore the Holm denominator (e.g., 28 for ELSA) reflects the full family of tested pathways and not just the rows displayed.

**Table S4. Care-receipt moderation of the partner effect: joint Wald F test of the two partner × care-category interactions per APIM transition, by cohort.**

| **Transition** | **Outcome** | ***n*** | **Main partner β (*p*)** | **Joint *F*** | **Joint *p*** | ***p* FDR** | ***p* Holm** |
| --- | --- | --- | --- | --- | --- | --- | --- |
| ***CHARLS*** | | | | | | | |
| W1→W2 | M | 2,894 | 0.059 (< 0.001) | 0.708 | 0.493 | 0.854 | 1.000 |
| W1→W2 | F | 2,877 | 0.073 (0.002) | 0.403 | 0.668 | 0.854 | 1.000 |
| W2→W3 | M | 2,858 | 0.074 (< 0.001) | 0.782 | 0.458 | 0.854 | 1.000 |
| W2→W3 | F | 2,839 | 0.084 (0.004) | 0.158 | 0.854 | 0.854 | 1.000 |
| W3→W4 | M | 2,747 | 0.080 (< 0.001) | 0.236 | 0.790 | 0.854 | 1.000 |
| W3→W4 | F | 2,831 | 0.107 (< 0.001) | 0.533 | 0.587 | 0.854 | 1.000 |
| W4→W5 | M | 2,344 | 0.022 (0.267) | 0.840 | 0.432 | 0.854 | 1.000 |
| W4→W5 | F | 2,275 | 0.075 (0.010) | 1.423 | 0.241 | 0.854 | 1.000 |
| ***HRS*** | | | | | | | |
| W1→W2 | M | 2,145 | −0.006 (.737) | 3.386 | .034 | .119 | .374 |
| W1→W2 | F | 2,153 | 0.056 (.030) | 4.368 | .013 | .060 | .153 |
| W2→W3 | M | 2,128 | 0.041 (.017) | 0.042 | .959 | .959 | 1.000 |
| W2→W3 | F | 2,162 | 0.067 (.005) | 0.770 | .463 | .810 | 1.000 |
| W3→W4 | M | 1,959 | 0.004 (.854) | 1.865 | .155 | .366 | 1.000 |
| W3→W4 | F | 2,025 | 0.001 (.980) | 0.256 | .774 | .903 | 1.000 |
| W4→W5 | M | 1,704 | 0.032 (.166) | 0.898 | .407 | .810 | 1.000 |
| W4→W5 | F | 1,819 | 0.045 (.091) | 0.546 | .579 | .811 | 1.000 |
| W5→W6 | M | 1,410 | 0.013 (.620) | 1.855 | .157 | .366 | 1.000 |
| W5→W6 | F | 1,479 | 0.048 (.115) | 5.932 | .003 | .019 | .035 |
| W6→W7 | M | 1,114 | −0.027 (.343) | 0.137 | .872 | .939 | 1.000 |
| W6→W7 | F | 1,179 | 0.061 (.069) | 7.319 | < 0.001 | .010 | .010 |
| W7→W8 | M | 880 | 0.048 (.141) | 0.584 | .558 | .811 | 1.000 |
| W7→W8 | F | 932 | −0.031 (.449) | 0.423 | .655 | .834 | 1.000 |
| ***ELSA*** | | | | | | | |
| W1→W2 | M | 1,686 | 0.012 (0.601) | 1.447 | 0.236 | 0.335 | 1.000 |
| W1→W2 | F | 1,562 | −0.003 (0.943) | 1.415 | 0.243 | 0.335 | 1.000 |
| W2→W3 | M | 1,632 | −0.013 (0.548) | 0.530 | 0.589 | 0.589 | 1.000 |
| W2→W3 | F | 1,504 | 0.063 (0.086) | 1.381 | 0.252 | 0.335 | 1.000 |
| W3→W4 | M | 1,382 | 0.054 (0.016) | 3.190 | 0.041 | 0.222 | 0.622 |
| W3→W4 | F | 1,310 | 0.103 (0.009) | 1.836 | 0.160 | 0.284 | 1.000 |
| W4→W5 | M | 1,233 | 0.004 (0.898) | 2.266 | 0.104 | 0.238 | 1.000 |
| W4→W5 | F | 1,160 | 0.098 (0.023) | 1.018 | 0.362 | 0.404 | 1.000 |
| W5→W6 | M | 1,128 | 0.071 (0.006) | 0.973 | 0.378 | 0.404 | 1.000 |
| W5→W6 | F | 1,055 | 0.041 (0.311) | 2.493 | 0.083 | 0.222 | 0.914 |
| W6→W7 | M | 967 | −0.011 (0.696) | 1.897 | 0.151 | 0.284 | 1.000 |
| W6→W7 | F | 926 | 0.081 (0.086) | 2.709 | 0.067 | 0.222 | 0.873 |
| W7→W8 | M | 810 | 0.045 (0.153) | 1.103 | 0.332 | 0.404 | 1.000 |
| W7→W8 | F | 778 | 0.101 (0.055) | 4.640 | 0.010 | 0.159 | 0.159 |
| W8→W9 | M | 670 | 0.102 (0.003) | 2.864 | 0.058 | 0.222 | 0.808 |
| W8→W9 | F | 645 | 0.144 (0.030) | 2.633 | 0.073 | 0.222 | 0.873 |

***Note.*** Joint Wald F-test for the two partner × care-category interactions in the care-moderated APIM (reference category: “no care needed”). “Main partner β” is the unstandardized partner-effect coefficient in the no-care-needed reference category, on the within-cohort z-standardized depression metric. FDR (Benjamini–Hochberg) and Holm adjustments are applied within cohort across the eight transition-by-outcome cells. No transition reached adjusted significance in CHARLS or ELSA.

**Table S5. Bivariate Random-Intercept Cross-Lagged Panel Model: model-fit indices, time-invariant within-person cross-lagged paths, and between-person random-intercept structure, by cohort.**

***Panel A. Model-fit indices.***

| **Model** | **χ²** | ***df*** | **CFI** | **TLI** | **RMSEA** | **SRMR** | **AIC** | **BIC** | ***n*** |
| --- | --- | --- | --- | --- | --- | --- | --- | --- | --- |
| ***CHARLS*** | | | | | | | | | |
| Unconstrained Hamaker | 84.18 | 21 | .994 | .987 | .029 | .020 | 77,495 | 77,767 | 3,532 |
| Time-invariant Hamaker | 158.22 | 33 | .988 | .984 | .033 | .032 | 77,545 | 77,743 | 3,532 |
| Simplified RI-CLPM | 77.76 | 21 | .995 | .988 | .028 | .019 | 77,489 | 77,760 | 3,532 |
| Traditional CLPM | 1988.56 | 36 | .813 | .766 | .124 | .130 | 79,369 | 79,548 | 3,532 |
| ***HRS*** | | | | | | | | | |
| Unconstrained Hamaker | 271.21 | 81 | .982 | .973 | .032 | .043 | 79,108 | 79,516 | 2,332 |
| Time-invariant Hamaker | 429.59 | 105 | .969 | .965 | .036 | .056 | 79,218 | 79,489 | 2,332 |
| Simplified RI-CLPM | 254.70 | 81 | .984 | .976 | .030 | .030 | 79,091 | 79,500 | 2,332 |
| Traditional CLPM | 2626.46 | 108 | .761 | .735 | .100 | .168 | 81,409 | 81,662 | 2,332 |
| ***ELSA*** | | | | | | | | | |
| Unconstrained Hamaker | 353.53 | 109 | 0.973 | 0.962 | 0.034 | 0.041 | 65,345 | 65,789 | 1,891 |
| Time-invariant Hamaker | 466.31 | 137 | 0.963 | 0.959 | 0.036 | 0.057 | 65,402 | 65,691 | 1,891 |
| Simplified RI-CLPM | 366.25 | 109 | 0.971 | 0.960 | 0.035 | 0.037 | 65,358 | 65,802 | 1,891 |
| Traditional CLPM | 2173.78 | 140 | 0.772 | 0.751 | 0.088 | 0.174 | 67,104 | 67,375 | 1,891 |

***Panel B. Time-invariant within-person cross-lagged paths.***

| **Path** | **Interpretation** | **β (SE)** | ***z*** | ***p*** | **Std. β** | **95% CI** |
| --- | --- | --- | --- | --- | --- | --- |
| ***CHARLS*** | | | | | | |
| aM | Male autoregressive (cM → cM) | 0.077 (0.018) | 4.40 | < 0.001 | .077 | [0.043, 0.111] |
| aF | Female autoregressive (cF → cF) | 0.085 (0.015) | 5.53 | < 0.001 | .086 | [0.055, 0.116] |
| xMF | Male → Female cross-lagged path (cM → cF) | 0.007 (0.015) | 0.46 | .645 | .006 | [−0.022, 0.036] |
| xFM | Female → Male cross-lagged path (cF → cM) | 0.022 (0.012) | 1.84 | .065 | .025 | [−0.001, 0.045] |
| ***HRS*** | | | | | | |
| aM | Male autoregressive (cM → cM) | 0.162 (0.024) | 6.72 | < 0.001 | 0.158 | [0.115, 0.209] |
| aF | Female autoregressive (cF → cF) | 0.132 (0.019) | 6.94 | < 0.001 | 0.130 | [0.095, 0.170] |
| xMF | Male → Female cross-lagged path (cM → cF) | 0.028 (0.019) | 1.44 | .151 | 0.023 | [−0.010, 0.066] |
| xFM | Female → Male cross-lagged path (cF → cM) | 0.014 (0.013) | 1.05 | .292 | 0.016 | [−0.012, 0.039] |
| ***ELSA*** | | | | | | |
| aM | Male autoregressive (cM → cM) | 0.193 (0.021) | 9.01 | < 0.001 | .194 | [0.151, 0.235] |
| aF | Female autoregressive (cF → cF) | 0.153 (0.018) | 8.54 | < 0.001 | .152 | [0.118, 0.188] |
| xMF | Male → Female cross-lagged path (cM → cF) | 0.064 (0.018) | 3.48 | < 0.001 | .053 | [0.028, 0.100] |
| xFM | Female → Male cross-lagged path (cF → cM) | 0.026 (0.012) | 2.17 | 0.030 | .031 | [0.003, 0.049] |

***Panel C. Between-person random-intercept variances and covariance.***

| **Parameter** | **Interpretation** | **Estimate** | **SE** | ***p*** | **Std. (ρ)** |
| --- | --- | --- | --- | --- | --- |
| ***CHARLS*** | | | | | |
| RIM ~~ RIF | Male–female RI covariance | 0.224 | 0.012 | < 0.001 | .523 |
| RIM ~~ RIM | Male RI variance | 0.370 | 0.014 | < 0.001 | 1.000 |
| RIF ~~ RIF | Female RI variance | 0.495 | 0.017 | < 0.001 | 1.000 |
| ***HRS*** | | | | | |
| RIM ~~ RIF | Male–female RI covariance | 0.149 | 0.019 | < 0.001 | 0.305 |
| RIM ~~ RIM | Male RI variance | 0.448 | 0.031 | < 0.001 | 1.000 |
| RIF ~~ RIF | Female RI variance | 0.534 | 0.032 | < 0.001 | 1.000 |
| ***ELSA*** | | | | | |
| RIM ~~ RIF | Male–female RI covariance | 0.171 | 0.018 | < 0.001 | .416 |
| RIM ~~ RIM | Male RI variance | 0.331 | 0.025 | < 0.001 | 1.000 |
| RIF ~~ RIF | Female RI variance | 0.512 | 0.031 | < 0.001 | 1.000 |

***Note.*** Panel A: all models were estimated by full-information maximum likelihood with robust (MLR) standard errors using lavaan in R. CFI ≥ .95, RMSEA ≤ .06 and SRMR ≤ .08 indicate excellent model-data fit. Panel B: time-invariant within-person cross-lagged paths from the Hamaker (2015) RI-CLPM after removing the four random intercepts that absorb between-couple stable similarity. Panel C: between-person random-intercept variances and the across-spouse RI covariance / standardized correlation, capturing stable between-couple similarity in depressive symptoms.

**Table S6. Bivariate Random-Intercept Cross-Lagged Panel Model: multiple-imputation-pooled within-person cross-lagged paths, by cohort.**

| **Path** | **Interpretation** | ***m*** | **β (SE)** | ***p*** | **FMI** | **Std. β** | **95% CI** |
| --- | --- | --- | --- | --- | --- | --- | --- |
| ***CHARLS*** | | | | | | | |
| aM | Male autoregressive (cM → cM) | 20 | 0.085 (0.017) | < 0.001 | .213 | .098 | [0.052, 0.118] |
| aF | Female autoregressive (cF → cF) | 20 | 0.090 (0.015) | < 0.001 | .196 | .102 | [0.061, 0.120] |
| xMF | Male → Female cross-lagged path | 20 | 0.013 (0.014) | .358 | .214 | .013 | [−0.015, 0.042] |
| xFM | Female → Male cross-lagged path | 20 | 0.023 (0.012) | .062 | .282 | .029 | [−0.001, 0.046] |
| ***HRS*** | | | | | | | |
| aM | Male autoregressive (cM → cM) | 20 | 0.230 (0.019) | < 0.001 | 0.196 | 0.234 | [0.193, 0.267] |
| aF | Female autoregressive (cF → cF) | 20 | 0.159 (0.016) | < 0.001 | 0.180 | 0.161 | [0.128, 0.191] |
| xMF | Male → Female cross-lagged path | 20 | 0.061 (0.016) | < 0.001 | 0.238 | 0.053 | [0.030, 0.092] |
| xFM | Female → Male cross-lagged path | 20 | 0.044 (0.011) | < 0.001 | 0.273 | 0.053 | [0.022, 0.067] |
| ***ELSA*** | | | | | | | |
| aM | Male autoregressive (cM → cM) | 20 | 0.229 (0.017) | < 0.001 | .209 | .225 | [0.196, 0.261] |
| aF | Female autoregressive (cF → cF) | 20 | 0.164 (0.015) | < 0.001 | .212 | .167 | [0.135, 0.193] |
| xMF | Male → Female cross-lagged path | 20 | 0.087 (0.015) | < 0.001 | .289 | .073 | [0.057, 0.117] |
| xFM | Female → Male cross-lagged path | 20 | 0.051 (0.010) | < 0.001 | .243 | .061 | [0.031, 0.071] |

***Note.*** RI-CLPM cross-lags estimated separately in each of m = 20 imputed datasets handed off from the Python pipeline and pooled by Rubin’s rules. m = number of imputed datasets that converged. FMI = fraction of missing information. Standardized β is the cross-imputation mean of the completely standardized solution.

**Table S7. Sex-distinguishability tests at each wave transition: joint Wald test of role × actor and role × partner interactions in long-format dyadic regressions, by cohort.**

| **Transition** | **Couples (*n*)** | **Female − male partner difference** | **Joint *F*** | **Joint *p*** | ***p* FDR** | ***p* Holm** | **Distinguishable at FDR 0.05?** |
| --- | --- | --- | --- | --- | --- | --- | --- |
| ***CHARLS*** | | | | | | | |
| W1→W2 (2011–13) | 3,021 | 0.004 | 1.041 | 0.353 | 0.659 | 1.000 | No |
| W2→W3 (2013–15) | 2,909 | 0.012 | 0.706 | 0.494 | 0.659 | 1.000 | No |
| W3→W4 (2015–18) | 3,022 | 0.023 | 0.403 | 0.669 | 0.669 | 1.000 | No |
| W4→W5 (2018–20) | 2,611 | 0.020 | 0.953 | 0.386 | 0.659 | 1.000 | No |
| ***HRS*** | | | | | | | |
| W1→W2 (06–08) | 2,177 | +0.060 | 1.846 | .158 | .324 | 0.948 | No |
| W2→W3 (08–10) | 2,193 | +0.043 | 1.394 | .248 | .348 | 0.948 | No |
| W3→W4 (10–12) | 2,115 | +0.000 | 1.806 | .165 | .324 | 0.948 | No |
| W4→W5 (12–14) | 1,908 | +0.002 | 1.688 | .185 | .324 | 0.948 | No |
| W5→W6 (14–16) | 1,577 | +0.030 | 0.597 | .551 | .642 | 1.000 | No |
| W6→W7 (16–18) | 1,265 | +0.125 | 3.715 | .025 | .172 | 0.175 | No |
| W7→W8 (18–20) | 1,004 | −0.026 | 0.148 | .862 | .862 | 1.000 | No |
| ***ELSA*** | | | | | | | |
| W1→W2 (02–04) | 1,777 | 0.012 | 0.056 | 0.946 | 0.946 | 1.000 | No |
| W2→W3 (04–06) | 1,715 | 0.072 | 3.117 | 0.045 | 0.225 | 0.356 | No |
| W3→W4 (06–08) | 1,521 | 0.042 | 0.583 | 0.558 | 0.745 | 1.000 | No |
| W4→W5 (08–10) | 1,352 | 0.105 | 2.886 | 0.056 | 0.225 | 0.393 | No |
| W5→W6 (10–12) | 1,224 | −0.031 | 2.242 | 0.107 | 0.284 | 0.640 | No |
| W6→W7 (12–14) | 1,061 | 0.084 | 1.651 | 0.192 | 0.385 | 0.962 | No |
| W7→W8 (14–16) | 898 | −0.006 | 0.140 | 0.869 | 0.946 | 1.000 | No |
| W8→W9 (16–18) | 749 | 0.088 | 0.699 | 0.498 | 0.745 | 1.000 | No |

***Note.*** Sex distinguishability was assessed at each wave transition by a joint Wald F-test of the role × actor and role × partner interactions in long-format dyadic regressions with cluster-robust standard errors (clustered on couple). The “female − male partner difference” is the wife − husband contrast on the partner-effect coefficient. *p* FDR and Holm adjustments are applied within cohort across transitions. No transition rejected the sex-symmetric null at Holm-adjusted α = 0.05 in any cohort.

**Table S8. Reporting-bias robustness analyses: four-variable Random-Intercept Cross-Lagged Panel Model with IADL functional status, Satorra–Bentler scaled difference test, and bivariate-versus-four-variable attenuation diagnostic, by cohort.**

***Panel A. Four-variable RI-CLPM (IADL sensitivity model): time-invariant within-person cross-construct paths between depressive symptoms and IADL.***

| **Path** | **Description** | **Outcome ← Predictor** | **β (SE)** | **95% CI** | ***p*** | **Std. β** | **Sig.** |
| --- | --- | --- | --- | --- | --- | --- | --- |
| ***CHARLS*** | | | | | | | |
| bMdMf | Actor: own function → own depression | Male dep ← Male IADL | 0.051 (0.021) | [0.010, 0.092] | .014 | .062 | * |
| bMdFf | Partner: spouse function → own dep | Male dep ← Female IADL | −0.001 (0.011) | [−0.022, 0.021] | .953 | −.001 |  |
| bMfMd | Actor: own dep → own function | Male IADL ← Male dep | 0.039 (0.024) | [−0.008, 0.085] | .100 | .029 |  |
| bMfFd | Partner: spouse dep → own function | Male IADL ← Female dep | −0.011 (0.017) | [−0.045, 0.022] | .500 | −.010 |  |
| bFdMf | Partner: spouse function → own dep | Female dep ← Male IADL | 0.001 (0.016) | [−0.031, 0.033] | .948 | .001 |  |
| bFdFf | Actor: own function → own depression | Female dep ← Female IADL | 0.024 (0.012) | [0.000, 0.048] | .053 | .030 |  |
| bFfMd | Partner: spouse dep → own function | Female IADL ← Male dep | 0.012 (0.022) | [−0.030, 0.054] | .578 | .008 |  |
| bFfFd | Actor: own dep → own function | Female IADL ← Female dep | 0.038 (0.020) | [−0.001, 0.077] | .058 | .029 |  |
| ***HRS*** | | | | | | | |
| bMdMf | Actor: own function → own depression | Male dep ← Male IADL | 0.079 (0.012) | [0.056, 0.103] | < 0.001 | 0.188 | *** |
| bMdFf | Partner: spouse function → own dep | Male dep ← Female IADL | 0.049 (0.009) | [0.031, 0.066] | < 0.001 | 0.117 | *** |
| bMfMd | Actor: own dep → own function | Male IADL ← Male dep | 0.165 (0.035) | [0.097, 0.234] | < 0.001 | 0.061 | *** |
| bMfFd | Partner: spouse dep → own function | Male IADL ← Female dep | 0.055 (0.022) | [0.012, 0.098] | .012 | 0.024 | * |
| bFdMf | Partner: spouse function → own dep | Female dep ← Male IADL | 0.044 (0.008) | [0.028, 0.059] | < 0.001 | 0.090 | *** |
| bFdFf | Actor: own function → own depression | Female dep ← Female IADL | 0.085 (0.012) | [0.062, 0.109] | < 0.001 | 0.179 | *** |
| bFfMd | Partner: spouse dep → own function | Female IADL ← Male dep | 0.062 (0.027) | [0.008, 0.116] | .025 | 0.022 | * |
| bFfFd | Actor: own dep → own function | Female IADL ← Female dep | 0.106 (0.025) | [0.057, 0.154] | < 0.001 | 0.045 | *** |
| ***ELSA*** | | | | | | | |
| bMdMf | Actor: own function → own depression | Male dep ← Male IADL | 0.079 (0.013) | [0.053, 0.104] | < 0.001 | .181 | *** |
| bMdFf | Partner: spouse function → own dep | Male dep ← Female IADL | 0.063 (0.012) | [0.040, 0.086] | < 0.001 | .126 | *** |
| bMfMd | Actor: own dep → own function | Male IADL ← Male dep | 0.121 (0.029) | [0.065, 0.178] | < 0.001 | .049 | *** |
| bMfFd | Partner: spouse dep → own function | Male IADL ← Female dep | 0.032 (0.019) | [−0.005, 0.068] | .089 | .015 |  |
| bFdMf | Partner: spouse function → own dep | Female dep ← Male IADL | 0.040 (0.010) | [0.022, 0.059] | < 0.001 | .078 | *** |
| bFdFf | Actor: own function → own depression | Female dep ← Female IADL | 0.067 (0.020) | [0.029, 0.105] | .001 | .113 | *** |
| bFfMd | Partner: spouse dep → own function | Female IADL ← Male dep | 0.055 (0.029) | [−0.001, 0.112] | .056 | .025 |  |
| bFfFd | Actor: own dep → own function | Female IADL ← Female dep | 0.073 (0.025) | [0.024, 0.121] | .003 | .040 | ** |

***Panel B. Joint Satorra–Bentler likelihood-ratio test of the four constrained depression → function paths.***

| **Cohort** | **Specification** | **Δχ²** | ***df*** | ***p*** | **Verdict** |
| --- | --- | --- | --- | --- | --- |
| CHARLS | ADL | 17.37 | 4 | .002 | d→f paths jointly significant; bidirectional is correct spec |
| CHARLS | IADL | 6.27 | 4 | .180 | d→f paths not jointly significant; simpler f→d-only defensible |
| HRS | ADL | 28.60 | 4 | < 0.001 | d→f paths jointly highly significant; bidirectional is correct spec |
| HRS | IADL | 58.31 | 4 | < 0.001 | d→f paths jointly highly significant; bidirectional is correct spec |
| ELSA | ADL | 58.21 | 4 | < 0.001 | d→f paths jointly highly significant; bidirectional is correct spec |
| ELSA | IADL | 40.22 | 4 | < 0.001 | d→f paths jointly highly significant; bidirectional is correct spec |

***Panel C. Per-path comparison of the four function → depression paths between the bidirectional and forward-only specifications (ADL primary model).***

| **Path** | **Description** | **β bidirectional (*p*)** | **β f→d-only (*p*)** | **% change** | **CI overlap** | **Verdict tier** |
| --- | --- | --- | --- | --- | --- | --- |
| ***CHARLS*** | | | | | | |
| bMdMf | M dep ← M ADL | 0.060 (.004) | 0.043 (.012) | −28.9% | Yes | (b) Mild shift |
| bMdFf | M dep ← F ADL | −0.001 (.952) | −0.004 (.710) | +440.5% | Yes | (0) Null both |
| bFdMf | F dep ← M ADL | −0.007 (.656) | −0.003 (.831) | −60.5% | Yes | (0) Null both |
| bFdFf | F dep ← F ADL | 0.025 (.068) | 0.014 (.222) | −42.0% | Yes | (b) Mild shift |
| ***HRS*** | | | | | | |
| bMdMf | M dep ← M ADL | 0.086 (< 0.001) | 0.070 (< 0.001) | −18.6% | Yes | (b) Mild shift |
| bMdFf | M dep ← F ADL | 0.043 (< 0.001) | 0.034 (< 0.001) | −21.9% | Yes | (b) Mild shift |
| bFdMf | F dep ← M ADL | 0.057 (< 0.001) | 0.054 (< 0.001) | −4.7% | Yes | (a) Robust |
| bFdFf | F dep ← F ADL | 0.083 (< 0.001) | 0.067 (< 0.001) | −19.1% | Yes | (b) Mild shift |
| ***ELSA*** | | | | | | |
| bMdMf | M dep ← M ADL | 0.100 (< 0.001) | 0.075 (< 0.001) | −25.5% | Yes | (b) Mild shift |
| bMdFf | M dep ← F ADL | 0.043 (0.014) | 0.041 (0.009) | −3.1% | Yes | (a) Robust |
| bFdMf | F dep ← M ADL | 0.049 (0.007) | 0.037 (0.031) | −24.6% | Yes | (b) Mild shift |
| bFdFf | F dep ← F ADL | 0.049 (0.021) | 0.028 (0.114) | −41.5% | Yes | (b) Mild shift |

***Panel D. Bivariate-versus-four-variable attenuation diagnostic for the depression–depression paths.***

| **Path** | **Spec.** | **β (2-var)** | **95% CI (2-var)** | **β (4-var)** | **95% CI (4-var)** | **% change** | **CI overlap** |
| --- | --- | --- | --- | --- | --- | --- | --- |
| ***CHARLS*** | | | | | | | |
| Male → Female dep-dep cross-lag | ADL | 0.007 | [−0.022, 0.036] | 0.013 | [−0.017, 0.043] | +94.3% | Yes |
| Female → Male dep-dep cross-lag | ADL | 0.022 | [−0.001, 0.045] | 0.020 | [−0.004, 0.044] | −9.1% | Yes |
| Male dep autoregressive | ADL | 0.077 | [0.043, 0.111] | 0.085 | [0.049, 0.122] | +10.8% | Yes |
| Female dep autoregressive | ADL | 0.085 | [0.055, 0.116] | 0.085 | [0.054, 0.116] | −0.2% | Yes |
| Male → Female dep-dep cross-lag | IADL | 0.007 | [−0.022, 0.036] | 0.011 | [−0.018, 0.040] | +57.1% | Yes |
| Female → Male dep-dep cross-lag | IADL | 0.022 | [−0.001, 0.045] | 0.021 | [−0.003, 0.045] | −4.5% | Yes |
| Male dep autoregressive | IADL | 0.077 | [0.043, 0.111] | 0.082 | [0.046, 0.118] | +6.5% | Yes |
| Female dep autoregressive | IADL | 0.085 | [0.055, 0.116] | 0.087 | [0.056, 0.118] | +2.4% | Yes |
| ***HRS*** | | | | | | | |
| Male → Female dep-dep cross-lag | ADL | 0.028 | [−0.010, 0.066] | 0.030 | [−0.008, 0.069] | +8.9% | Yes |
| Female → Male dep-dep cross-lag | ADL | 0.014 | [−0.012, 0.039] | 0.015 | [−0.010, 0.041] | +10.9% | Yes |
| Male dep autoregressive | ADL | 0.162 | [0.115, 0.209] | 0.168 | [0.120, 0.217] | +3.9% | Yes |
| Female dep autoregressive | ADL | 0.132 | [0.095, 0.170] | 0.123 | [0.085, 0.160] | −7.5% | Yes |
| Male → Female dep-dep cross-lag | IADL | 0.028 | [−0.010, 0.066] | 0.033 | [−0.005, 0.070] | +17.4% | Yes |
| Female → Male dep-dep cross-lag | IADL | 0.014 | [−0.012, 0.039] | 0.012 | [−0.012, 0.037] | −8.1% | Yes |
| Male dep autoregressive | IADL | 0.162 | [0.115, 0.209] | 0.166 | [0.119, 0.212] | +2.5% | Yes |
| Female dep autoregressive | IADL | 0.132 | [0.095, 0.170] | 0.129 | [0.091, 0.167] | −2.6% | Yes |
| ***ELSA*** | | | | | | | |
| Male → Female dep-dep cross-lag | ADL | 0.064 | [0.028, 0.100] | 0.061 | [0.025, 0.098] | −3.6% | Yes |
| Female → Male dep-dep cross-lag | ADL | 0.026 | [0.003, 0.049] | 0.023 | [−0.000, 0.046] | −12.6% | Yes |
| Male dep autoregressive | ADL | 0.193 | [0.151, 0.235] | 0.186 | [0.145, 0.227] | −3.8% | Yes |
| Female dep autoregressive | ADL | 0.153 | [0.118, 0.188] | 0.151 | [0.116, 0.186] | −1.2% | Yes |
| Male → Female dep-dep cross-lag | IADL | 0.064 | [0.028, 0.100] | 0.061 | [0.025, 0.098] | −4.7% | Yes |
| Female → Male dep-dep cross-lag | IADL | 0.026 | [0.003, 0.049] | 0.025 | [0.001, 0.048] | −4.6% | Yes |
| Male dep autoregressive | IADL | 0.193 | [0.151, 0.235] | 0.177 | [0.136, 0.218] | −8.4% | Yes |
| Female dep autoregressive | IADL | 0.153 | [0.118, 0.188] | 0.152 | [0.116, 0.188] | −0.5% | Yes |

***Note.*** Panel A: IADL substitutes for ADL within the four-variable RI-CLPM; the model structure is otherwise identical to the ADL primary model. Significance markers: **p* < 0.05, ***p* < 0.01, ****p* < 0.001 (un-adjusted within-model). Panel B: the bidirectional specification estimates all 16 transition paths, including four depression → function within-person paths that may be vulnerable to mood-coloured self-rated functional limitation reporting (state-dependent reporting bias). The f→d-only specification fixes those four paths at zero and re-estimates the function → depression paths. A significant Satorra–Bentler LRT indicates that the depression → function paths add explanatory power and the bidirectional specification is the correct primary analysis. Panel C: per-path bidirectional-vs-constrained comparisons for the four function → depression paths, with the four-tier robustness verdict (0 = null in both, a = robust, b = mild shift, c = material). Panel D: the four depression–depression within-person paths (xMF, xFM, aM, aF in the bivariate model corresponding to bWdHd, bHdWd, bHdHd, bWdWd in the four-variable model) are compared between the bivariate RI-CLPM and the four-variable RI-CLPM under both ADL (primary) and IADL (sensitivity) function specifications. % change = (β 4-var − β 2-var) / β 2-var × 100.

**Table S9. Missing-data mechanism: Little’s MCAR test and logistic-regression prediction of final-wave dropout, by cohort.**

**Panel A. Little’s test of the missing-completely-at-random (MCAR) assumption.**

| **Cohort** | **Couples (n)** | **CES–D variables (k)** | **χ²** | **df** | **p** | **Conclusion** |
| --- | --- | --- | --- | --- | --- | --- |
| ***CHARLS*** | 3,532 | 10 | 1100.5 | 990 | .008 | MCAR rejected |
| ***HRS*** | 2,332 | 16 | 5610.9 | 3,979 | < 0.001 | MCAR rejected |
| ***ELSA*** | 1,895 | 18 | 5133.0 | 4,030 | < 0.001 | MCAR rejected |

**Panel B. Logistic regression of final-wave dropout on baseline characteristics: odds ratio (95% CI).**

| **Baseline predictor** | **CHARLS** | | **HRS** | | **ELSA** | |
| --- | --- | --- | --- | --- | --- | --- |
|  | **Husbands** | **Wives** | **Husbands** | **Wives** | **Husbands** | **Wives** |
| ***Husband age*** | 1.10 (1.07–1.13)* | 1.05 (1.02–1.07)* | 1.09 (1.07–1.12)* | 1.02 (0.99–1.04) | 1.09 (1.06–1.12)* | 1.03 (1.00–1.05)* |
| ***Wife age*** | 0.99 (0.97–1.02) | 1.03 (1.01–1.06)* | 1.02 (0.99–1.04) | 1.08 (1.05–1.10)* | 1.00 (0.97–1.03) | 1.04 (1.02–1.07)* |
| ***Husband education*** | 0.80 (0.64–1.01) | 0.82 (0.65–1.03) | 0.80 (0.68–0.95)* | 1.01 (0.86–1.19) | 0.93 (0.77–1.11) | 0.99 (0.83–1.18) |
| ***Wife education*** | 1.33 (0.96–1.85) | 1.00 (0.71–1.42) | 0.95 (0.79–1.14) | 0.89 (0.74–1.06) | 0.78 (0.65–0.95)* | 0.72 (0.60–0.87)* |
| ***Husband baseline depression*** | 1.03 (0.93–1.15) | 1.00 (0.90–1.12) | 0.95 (0.85–1.07) | 0.98 (0.88–1.10) | 1.15 (1.00–1.33)* | 1.08 (0.94–1.23) |
| ***Wife baseline depression*** | 1.11 (1.01–1.23)* | 1.08 (0.98–1.19) | 1.01 (0.91–1.11) | 1.13 (1.03–1.24)* | 0.98 (0.88–1.09) | 0.93 (0.83–1.03) |
| ***Husband baseline ADL*** | 1.05 (0.93–1.19) | 0.91 (0.79–1.04) | 1.05 (0.83–1.33) | 1.12 (0.90–1.40) | 1.06 (0.88–1.28) | 0.98 (0.83–1.17) |
| ***Wife baseline ADL*** | 0.95 (0.85–1.06) | 0.99 (0.89–1.11) | 1.15 (0.93–1.42) | 1.07 (0.88–1.31) | 0.94 (0.78–1.13) | 1.15 (0.95–1.37) |
| ***Husband baseline IADL*** | 1.18 (1.05–1.33)* | 1.02 (0.90–1.15) | 1.14 (0.81–1.61) | 0.95 (0.69–1.31) | 1.75 (1.07–2.86)* | 0.89 (0.63–1.28) |
| ***Wife baseline IADL*** | 1.02 (0.92–1.12) | 1.21 (1.10–1.33)* | 1.07 (0.75–1.52) | 1.57 (1.09–2.24)* | 1.00 (0.71–1.40) | 0.98 (0.72–1.35) |
| ***Husband baseline self-rated health*** | 1.08 (0.97–1.20) | 0.96 (0.86–1.07) | 0.73 (0.65–0.81)* | 1.00 (0.90–1.11) | 1.15 (1.02–1.30)* | 1.08 (0.96–1.22) |
| ***Wife baseline self-rated health*** | 0.88 (0.79–0.98)* | 1.04 (0.93–1.16) | 0.94 (0.84–1.05) | 0.80 (0.71–0.89)* | 1.10 (0.97–1.24) | 1.07 (0.95–1.21) |

***Note.*** Panel A reports Little’s test of the missing-completely-at-random (MCAR) assumption applied to the repeated CES–D depressive-symptom measures (both spouses at all waves: 10 variables in CHARLS, 16 in HRS, 18 in ELSA); k is the number of such variables and a statistically significant test indicates that the data are not MCAR. Panel B reports, for each cohort, two separate logistic regressions in which the outcome is dropout, defined as a missing CES–D observation at the cohort’s final wave (CHARLS wave 5, 2020; HRS wave 8, 2020; ELSA wave 9, 2018). Each model regresses dropout on both spouses’ baseline (wave 1) depressive symptoms, ADL limitations, IADL limitations, self-rated health, age, and education; cells report the odds ratio with its 95% confidence interval for a one-unit increase in the predictor, estimated among couples with complete baseline predictor data. An asterisk denotes p *< 0.05*. In all three cohorts the MCAR null was rejected, and final-wave dropout was significantly predicted by one or more observed baseline characteristics in every cohort and for both spouses—most consistently older age, and in several models also higher baseline depressive symptoms, more IADL limitations, self-rated health, and lower education. Because attrition is systematically related to observed baseline data rather than completely random, it is incompatible with an MCAR mechanism and consistent with a missing-at-random (MAR) mechanism conditional on these observed variables; the full-information maximum likelihood and multiple-imputation analyses used throughout are valid under MAR. A missing-not-at-random component cannot be excluded and is acknowledged in the main-text Limitations.

**Table S10. Cross-cohort multi-group confirmatory factor analysis (MGCFA) of the six common CES-D items: model-fit indices under both CHARLS-binarisation thresholds.**

| **Model** | **Threshold** | **CFI** | **TLI** | **RMSEA** | **SRMR** | **ΔCFI** | **ΔRMSEA** |
| --- | --- | --- | --- | --- | --- | --- | --- |
| Configural | Strict (≥ 3) | 0.981 | 0.969 | 0.052 | 0.046 | — | — |
| Configural | Lenient (≥ 2) | 0.981 | 0.969 | 0.055 | 0.047 | — | — |
| Full metric | Strict (≥ 3) | 0.959 | 0.950 | 0.065 | 0.080 | −0.022 | +0.013 |
| Full metric | Lenient (≥ 2) | 0.971 | 0.965 | 0.058 | 0.067 | −0.010 | +0.003 |
| Partial metric (“Happy” free) | Strict (≥ 3) | 0.979 | 0.973 | 0.048 | 0.054 | −0.003 | −0.003 |
| Partial metric (“Happy” free) | Lenient (≥ 2) | 0.979 | 0.973 | 0.051 | 0.056 | −0.003 | −0.004 |

***Note.*** *Multi-group confirmatory factor analysis on the six items common to CHARLS, HRS, and ELSA (“Depressed,” “Effort,” “Restless sleep,” “Happy” positively keyed, “Lonely,” “Could not get going”), fitted in lavaan with the WLSMV estimator for binary indicators. HRS and ELSA items are natively binary; the CHARLS Likert responses were dichotomised at two thresholds (strict, ≥ 3 → 1; lenient, ≥ 2 → 1) to verify robustness. Analytic sample: 30,495 (CHARLS), 23,386 (ELSA), and 30,365 (HRS) person-wave observations. Partial metric invariance with only the positively keyed “Happy” loading freed meets the Cheung–Rensvold (2002) criteria (ΔCFI ≥ −.010 and ΔRMSEA ≤ .015 versus configural) under both binarisation thresholds, supporting comparability of structural coefficients across cohorts.*

**Table S11. Standardised factor loadings for the six common CES-D items in the configural MGCFA (strict binarisation threshold, CHARLS ≥ 3), by cohort.**

| **CES-D item** | **CHARLS** | **ELSA** | **HRS** | **Loading range** |
| --- | --- | --- | --- | --- |
| Depressed | 0.823 | 0.850 | 0.888 | 0.065 |
| Effort | 0.774 | 0.877 | 0.742 | 0.135 |
| Restless sleep | 0.565 | 0.497 | 0.549 | 0.068 |
| Happy (positively keyed) | 0.380 | 0.707 | 0.783 | 0.403 |
| Lonely | 0.772 | 0.659 | 0.717 | 0.113 |
| Could not get going | 0.803 | 0.814 | 0.697 | 0.117 |

***Note.*** *Standardised factor loadings from the configural model under the strict binarisation threshold. Loading range = cross-cohort maximum minus minimum. Five of the six items show closely matched loadings across cohorts (range 0.065–0.135). The positively keyed “Happy” item is the single source of non-invariance (range 0.403), consistent with prior CES-D harmonisation work showing that positively keyed items often function differently in East Asian samples (Van de Velde et al., 2010; Zhang et al., 2011). A polarity audit confirmed that the CHARLS “Happy” coding is correct (CHARLS r = 0.314; ELSA r = 0.365; HRS r = 0.421 between “Happy” and the sum of the remaining five items), so this non-invariance is substantive rather than an artefact of coding direction.*

**Supplementary Figures**

Each cohort panel of the supplementary figures listed below combines the three cohorts (CHARLS, HRS, ELSA) into a single multi-panel layout, with the cohort indicated by a label band above each panel. The figures themselves are produced by the analysis pipeline as PNG files (CHARLS, HRS, ELSA), and are intended for inclusion at the supplementary submission stage.

**Figure S1. Wave-by-wave evolution of CES-D depressive-symptom scores by gender in each cohort.**

***Note.*** Each cohort panel shows the mean CES-D score on the raw sum-score scale (Panel A) and after wave-1 z-standardisation (Panel B), plotted separately for males (blue circles) and females (orange squares) at each survey wave. Vertical whiskers and shaded ribbons denote 95% confidence intervals. Significance markers above each wave denote the between-gender difference at that wave (****p* < 0.001; ***p* < 0.01; **p* < 0.05; paired t-test, husband vs. wife within couples). The wave-by-wave gender gap, with females scoring systematically higher, is visible in all three cohorts but differs in absolute magnitude because CHARLS uses the 10-item CES-D (range 0–30) whereas HRS and ELSA use the 8-item CES-D (range 0–8).

**Figure S2. Wave-by-wave evolution of functional limitations (ADL, range 0–6; IADL, range 0–5) by gender in each cohort.**

***Note.*** Each cohort panel plots the mean count of ADL limitations (Panel A) and IADL limitations (Panel B), separately for males (blue) and females (orange) at each survey wave; whiskers and shaded ribbons denote 95% confidence intervals. The strong upward trend in functional limitations across all three cohorts is consistent with sample ageing across the observation window and motivates the inclusion of ADL/IADL as time-varying covariates in the four-variable RI-CLPM (Table 3 and Supplementary Table S8).

**Figure S3. Composition of spousal-care status across survey waves, by gender and cohort.**

***Note.*** Each cohort panel shows the proportion of couples in which the reference partner falls into each of four care-status categories at each survey wave: no care needed (green), care needed but not received (orange), care received from spouse (pink), and unknown/missing (grey). Across all three cohorts the no-care-needed share declines monotonically with sample ageing and the care-received share grows. The growth in the unknown share at later waves reflects loss-to-follow-up and item non-response, which differ in magnitude across cohorts (HRS shows the largest unknown-share growth, reaching about half of couples by W7–W8). This descriptive context motivates the care-status moderation analysis reported in Supplementary Table S4.

**Figure S4. Within-couple similarity of depressive symptoms over time, expressed as wave-specific Pearson correlation and intraclass correlation, by cohort.**

***Note.*** Each cohort panel plots the wave-specific within-couple Pearson r (solid line; left axis) and ICC (dashed line; right axis) computed on the standardised CES-D score within each survey wave. Shaded ribbons are 95% confidence intervals on the Pearson r. Within-couple similarity is positive and stable in HRS and ELSA across the observation window but appears to decline modestly in CHARLS from wave 1 to later waves, consistent with attrition of the most concordant couples and/or increasing within-couple variance with age. Numerical values appear in Supplementary Table S1.

**Figure S5. Wave-specific Actor–Partner Interdependence Model (APIM) cross-lagged coefficients for depressive symptoms, comparing complete-case (listwise) and multiple-imputation pooled estimates, by cohort.**

***Note.*** Each cohort panel shows wave-by-wave APIM standardised actor and partner coefficients for the male outcome (left sub-panel) and female outcome (right sub-panel). Solid markers are listwise-deletion estimates; open markers are estimates pooled across m = 20 multiply imputed datasets via Rubin’s rules. Whiskers are 95% confidence intervals. Listwise and MI-pooled estimates are visually overlapping at every transition in every cohort, consistent with the modest fractions of missing information at earlier waves and the larger but still tractable FMI values at the later-wave transitions (FMI range across cohorts 0.019–0.528; Supplementary Table S2), and with the conclusion that the APIM partner effects are robust to the choice of missingness handling.

**Figure S6. Confounding by stable between-couple similarity: traditional CLPM estimates versus RI-CLPM within-person estimates, by cohort.**

***Note.*** For each cohort the figure compares the autoregressive (top sub-panel) and cross-lagged (bottom sub-panel) coefficients estimated by the traditional time-invariant cross-lagged panel model (orange triangles) versus the Hamaker (2015) RI-CLPM that partitions stable between-person from time-varying within-person variance (green circles). In every cohort the CLPM autoregressive paths are biased upward by roughly an order of magnitude, and the CLPM cross-lags are also inflated. After within-person purification, the pattern differs by cohort: in CHARLS both cross-spouse within-person paths become non-distinguishable from zero (xMF and xFM attenuated by approximately 90% and 70%, respectively); in HRS both within-person paths are likewise non-distinguishable from zero under FIML (xMF attenuated by approximately 65%); whereas in ELSA both within-person cross-lags remain statistically distinguishable from zero with comparatively modest attenuation. This pattern motivates the use of the RI-CLPM as the primary analytic framework for within-couple depressive interdependence.
